# Characterizing candidate decompression rates for hypobaric hypoxic stunning of pigs. Part 1: Reflexive behavior and physiological responses

**DOI:** 10.3389/fvets.2022.1027878

**Published:** 2022-11-29

**Authors:** Jessica E. Martin, Emma M. Baxter, Jasmine M. Clarkson, Marianne Farish, Richard E. Clutton, Stephen N. Greenhalgh, Rachael Gregson, Dorothy E. F. McKeegan

**Affiliations:** ^1^The Royal (Dick) School of Veterinary Studies and The Roslin Institute, The University of Edinburgh, Edinburgh, United Kingdom; ^2^School of Natural and Environmental Sciences, Newcastle University, Newcastle upon Tyne, United Kingdom; ^3^Animal and Veterinary Sciences Research Group, Scotland's Rural College (SRUC), Edinburgh, United Kingdom; ^4^School of Biodiversity, One Health and Veterinary Medicine, College of Medical, Veterinary and Life Sciences, University of Glasgow, Glasgow, United Kingdom; ^5^The Wellcome Trust Critical Care Laboratory for Large Animals LARIF, The Roslin Institute, The University of Edinburgh, Edinburgh, United Kingdom

**Keywords:** low atmospheric pressure, slaughter, killing, swine, decompression, animal welfare

## Abstract

Alternatives to carbon dioxide (CO_2_) stunning for the commercial slaughter of pigs are urgently needed because there is robust evidence that exposing pigs to hypercapnic environments is associated with pain, fear, and distress. Hypobaric hypoxia (via gradual decompression, also known as Low Atmospheric Pressure Stunning or LAPS) has been validated in poultry as a humane option, but its potential to improve the welfare of pigs at slaughter is unknown. We investigated the potential of hypobaric hypoxia to reliably elicit a non-recovery state in anesthetized weaner-grower pigs within a commercially viable timeframe. We determined the effect of candidate decompression rates (40, 60, 80, 100 ms^−1^, at two cycle durations 480 s and 720 s) on a range of physiological and reflexive behavioral indicators of hypoxia and death. We found that the decompression rates tested caused a 100% death rate. As expected, the decompression rate had overarching effects on behavioral and physiological markers of hypoxia and death, with faster decompression rates resulting in shorter latencies to cardiac arrest and cessation of breathing. We observed a higher proportion of pigs displaying repeated and prolonged whole-body movements (likely indicative of convulsive activity) at higher frequencies when we applied the slowest decompression rate (40 ms^−1^) compared to all other rates. Since these responses may impact the carcass and meat quality, the slower rate of decompression (40 ms^−1^) should be excluded as a candidate decompression rate. Furthermore, given the marginal effects of decompression rate on physiological indicators of death and reflexive behavioral parameters, we also recommend that the fastest rate tested (100 ms^−1^) is excluded in further study on conscious pigs (to prevent conscious animals from being exposed to unnecessary faster decompression rates which may compromise animal welfare). This work represents a necessary proof of principle step and confirms the potential of gradual decompression for stunning purposes in pigs. Importantly, however, the data presented provide no information on the welfare outcomes associated with decompression in conscious pigs. Subsequent work should focus on the comprehensive welfare assessment of intermediate decompression rates to determine the potential of hypobaric hypoxia to provide a humane stunning method for pigs.

## Introduction

For the very large numbers of animals entering the food chain globally, protection of welfare at the time of slaughter is essential both in terms of regulatory requirements and also to provide reassurance to the general public who consume their products (1). In 2020, 1.4 billion pigs were slaughtered worldwide, with 256 million slaughtered in the EU, where controlled atmosphere stunning (CAS) with carbon dioxide (CO_2_) is the most frequently used approach (2). This reflects a progressive uptake of CO_2_ stunning (overtaking electrical stunning) because it offers major advantages: higher throughput and group-based pre-stun handling which reduces stress (3–6), as well as improvements to carcass and meat quality (7, 8). However, exposure to CO_2_, especially at high concentrations, is associated with significant welfare concerns, relating both to its nociceptive properties above threshold concentrations and because it is a potent stimulant of dyspnea and in particular air hunger (6, 9). Several studies characterizing the responses of pigs to hypercapnic environments provide convincing evidence of aversion (10–15) and as such the use of CO_2_ as a stunning agent for the commercial slaughter of pigs has become increasingly controversial (16, 17). Furthermore, urgent calls for the development of welfare-friendly alternatives (6, 16, 17) have recently been intensified in response to ongoing CO_2_ supply issues in Europe which have disrupted CAS operations with significant downstream animal welfare impacts (18). An alternative is yet to be identified and the limited scientific publications released in the past ~20 years have primarily focused on attempts to refine gas mixtures in combination with CO_2_ only (17, 19), with some studies suggesting a reduction in aversion (13, 20–22), while others have concluded no advantage (14, 23, 24). Even so, the use of some gas mixtures (e.g., argon and nitrogen) is challenging due to technical and economic factors which makes their commercial use problematic (6, 19, 25).

One potential alternative is hypobaric hypoxia (also known as Low Atmospheric Pressure Stunning or LAPS), whereby a non-recovery state is achieved by exposing animals to gradual decompression (25, 26). In this process, progressive decompression is applied to animals in a sealed chamber and the proportional decrease in oxygen partial pressure results in loss of consciousness and eventually death (27). This approach is attractive for pig slaughter since it would enable the retention of group handling and stunning, though throughput implications would depend on cycle length since LAPS is a batch (i.e., non-continuous) approach. Moreover, hypobaric hypoxia is not reliant on the use of gas supplies, potentially providing a logistical advantage. LAPS is garnering interest as a novel welfare-friendly stunning method, and recent extensive welfare assessments in poultry (26, 28–31) have underpinned its addition to EU Regulation 1099/2009 (32) as an approved method for stunning of broilers up to 4 kg and for depopulation purposes in 2018. Hypobaric hypoxia may enable the same welfare refinements observed when utilizing normobaric hypoxia for animal stunning, which involves the displacement of oxygen with an inert gas (e.g., argon or nitrogen) (10, 20). Pigs showed reduced behavioral responses indicative of aversion when exposed to argon-induced hypoxia in comparison with hypercapnic hypoxia induced by CO_2_ (10, 33). However, behavioral indicators of aversion were not fully abolished at high concentrations of argon (90%) (33). One reason for aversion could be “air hunger”—a potent and unpleasant experience associated with breathlessness (synonymous with “dyspnea”), described as the uncomfortable urge to breathe that develops progressively during a long breath hold (34). In human-subject reports, it is associated with anxiety, frustration, fear, and even panic (35). Both hypercapnia and hypoxia (the two primary induced states related to CAS systems for pigs and poultry) are potent stimuli for air hunger and yet this phenomenon has received little attention in these contexts and has been raised a neglected major welfare issue (9). It is not clear whether air hunger will be activated to the same extent by hypobaric and normobaric hypoxic environments, but both are likely to cause less respiratory discomfort than CO_2_ (36).

There is no peer-reviewed literature on hypobaric hypoxia in pigs, but there is an unpublished report and thesis examining its potential as an on-farm killing method for pre-weaned piglets (37, 38). Basic behavioral, physiological, and pathological measures demonstrated that death was achieved, but the findings revealed evidence of decompression sickness and worrying pathological and behavioral outcomes, with the majority of piglets displaying prolonged periods of gasping and vocalizations while conscious. These results likely relate to the prolonged duration of exposure (around 30 min) and the pathological findings are likely to be attributed to the extremely high-altitude equivalent conditions achieved (nearing Armstrong's line of 19,200 m altitude, where the International Standard Atmosphere (ISA) reaches <6.3 kPa resulting in the boiling temperature of water dropping to 37°C)—not necessary for hypoxic stunning (27). Bouwsema and Lines (25) discussed the feasibility of LAPS for pig stunning, and recommended ascent rates that are known to be unproblematic for humans, for example, an equivalent ascent from 0 to 13,716 m over 5 min (averaging 45.7 ms^−1^). For comparison, the LAPS cycle applied to poultry achieves a target altitude equivalent of 11,498 m in ~124 s [averaging a decompression rate of 127 ms^−1^, with minor fluctuations dependent on ambient environmental parameters (e.g., elevation, temperature, and relative humidity) (27)]. This results in pressure changes ranging from 1.7 kPas^−1^ (at the very start of the cycle) to 0.01 kPas^−1^ (during the hold phase). Given the significant anatomical and physiological differences between birds and mammals, there is a need to determine, from first principles, if hypobaric hypoxia (*via* gradual decompression) can provide an effective and non-aversive method of stunning for pigs.

This study aimed to explore the effectiveness of hypobaric hypoxia as a method for irreversibly stunning pigs and to identify candidate decompression parameters for potential further study. We characterized cardiac, respiratory, and reflexive behavioral responses to hypobaric hypoxia, and to protect the welfare, we worked on unconscious, terminally anesthetized pigs. Welfare assessment at slaughter usually encompasses the interpretation of physiological and reflexive behavioral responses (5, 15, 39, 40), and although we worked on anesthetized animals, such parameters still provide important data on the physiological and functional responses of body systems to hypoxia. As part of a systematic investigation of whether hypobaric hypoxia could be the basis of a humane, reliable, and efficient method of stunning for commercial pigs, we investigated four candidate decompression rates, all achieving the same final pressure. This study is Part 1 of a pair, with pathological findings from the same experiments published in Part 2 (41).

## Methods and materials

### Ethical approval

This study was conducted at the University of Edinburgh, following ethical approval from both the University of Edinburgh and SRUC Animal Welfare and Ethical Review Bodies (AWERBs, study approval refs: L325 and ED AE14-2018) and project license approval from the Home Office (PPL: PF5151DAF; Protocol 3). All work is reported to be fully compliant with the ARRIVE2.0 guidance. Daily monitoring of all animals was performed and no adverse effects were reported.

### Animals, housing, and husbandry

A total of sixty 10-week-old weaner-grower Large White (LW) x Landrace (LR) x Danish Duroc (DD) pigs (Rattlerow Farms Ltd, Suffolk, UK), balanced for sex and weighing approximately 30 Kg (mea*n* = 29.6 ± 0.5 Kg) were sourced from SRUC's pig unit and moved to the University of Edinburgh's research facility. All pigs were healthy and assessed as fit to travel before being recruited into the trial. Pigs were moved into familiar groups to reduce distress and aggression. On arrival, the pigs were housed in groups of six per pen in large pens [4 m x 4.6 m (18.4 m^2^)] bedded with deep straw and wood shavings, in climate-controlled rooms and lights on a timer (06:00–18:00). Pigs were provided with ad libitum access to water through adjustable height drinkers and dry pelleted feed (Ultra G200, ForFarmers, UK). Pens were supplemented with large dog chew toys to provide additional enrichment. Following transportation, pigs were given 48 h minimum to acclimatize to their new surroundings before experimental work started.

### Pre-stun anesthesia procedures and physiological monitoring

The pigs were anesthetized for the stun process which was maintained intravenously. Twelve hours before anesthesia, food was withdrawn from the group to prevent complications with intubation. On experimentally assigned stun days, pigs were gently moved in pairs into the anesthesiology room and housed in a pen according to treatment order where they were sedated. These pens (1.2 m x 1 m) had rubber matting on the floor (supplemented with straw) and semi-solid walls to prevent touching/interference from neighboring pigs in adjacent pens, but visual and olfactory contact was maintained.

Sedation was induced with azaperone 1 mgkg^−1^, ketamine 5 mgkg^−1^, midazolam 0.25 mgkg^−1^, and medetomidine 10 μgkg^−1^, combined in one syringe and administered *via* intramuscular injection to the brachiocephalic muscle in the neck. Sedation occurred within an average of 15.3 ± 0.8 min (range 12–31 min). Sedated pigs were lifted onto a table and, if required, isoflurane vaporized in oxygen (minimum F_I_O_2_ 0.3) and nitrous oxide was administered *via* face mask. An auricular vein was cannulated, after which anesthesia was maintained intravenously with an infusion of propofol at 0.2 mgkg^−1^minute^−1^. The trachea was intubated and the pigs spontaneously breathed oxygen *via* a Bain breathing system while physiological monitoring instrumentation was put in place. A multiparameter anesthesia monitor (DatexOhmeda (GE) S/5 Compact Anesthesia Monitor, US) was used to monitor several physiological variables [e.g., heart rate, respiration rate, and peripheral capillary oxygen saturation (SpO_2_)]. Disposable adhesive press-stud electrode sensors (Ambu Blue Sensor M-00-S/50, Ambu, UK) were applied to the pig's limbs and secured with adhesive tape allowing electrocardiogram (ECG) recording, and a pulse oximeter probe was clipped to the ear, allowing for detection of SpO_2_. Respiration was monitored by sidestream sampling of CO_2_ *via* a connector attached to the proximal end of the endotracheal tube. An adhesive bispectral index sensor (BIS, BIS™ Quatro sensors Aspect Medical Systems, USA) was placed on the head and connected to a BIS™ Complete 2-Channel Monitor (Medtronic, USA). The BIS sensor was further secured by a conforming bandage. Both the anesthetic and BIS monitor allowed continuous monitoring of the pig's physiological variables and evaluation of anesthetic depth. Additionally, disposable adhesive press-stud electrode sensors (Ambu Blue Sensor M-00-S/50, Ambu, UK) were placed on the thorax and connected to a custom-made battery-powered telemetry/logging device, housing a micro-SD memory cards (SanDisk 32GB, Maplin Electronics Ltd. Rotherham, UK), allowing continuous data logging of ECG waveforms at a sampling rate of 1 000 Hz (42). All pigs had an additional auricular venous cannula inserted to allow for rapid administration of substances (i.e., an overdose of barbiturates) for emergency euthanasia if required. On the basis of cranial reflex activity, including jaw tone, and other physiologic variables (including BIS), all pigs were judged to be at a surgical plane of anesthesia following anesthesia induction and until the decompression cycle was activated.

### Gradual decompression and the LAPS^®^ system

The LAPS^®^ system was developed by TechnoCatch LLC, USA, for the stunning of poultry (27). In brief, the system utilizes a large cylindrical chamber, with bespoke monitoring and control systems designed to operate desired decompression cycles and a separate vacuum pump. There are multiple sizes of chambers available as part of the LAPS^®^ system, all operating in the same way, but allowing for specific uses. In this study, we used a chamber developed for research purposes (2.5 m diameter, 3.7 m long), which allowed for an automated programmable logic controller (PLC) system, providing flexibility in decompression rate settings. The PLC recorded the chamber pressure (mmHg), temperature (°F), relative humidity (%), and atmospheric oxygen (%) at the start and during each executed cycle. The chamber had an automated hydraulic door, operated from the central PLC. Decompression cycles were pre-programmed to achieve the target decompression rates selected, but followed the same overall cycle profile as the commercial poultry settings, with two phases (26, 27). Phase 1 involved the vacuum chamber pressure being reduced from atmospheric pressure to an absolute vacuum of ~33 kPa (equivalent to 8,459 m); and the second phase (hold phase) involved modulation by a sliding gate valve, reducing the pumping speed *via* “choke flow” and slowing the decompression in the chamber to the final absolute vacuum of ~20 kPa (equivalent to 11,498 m). The length of each phase was dependent on the decompression rate selected for each treatment; however, the total cycle length was fixed to 720 s (12 min) or 480 s (8 min). The reduction in total pressure causes a synchronized reduction in oxygen partial pressure, and therefore a reduction in oxygen available to breathe. At the end of the cycle, the chamber is returned to atmospheric pressure over a fixed period of 60 s of recompression using a baffled air inlet. The LAPS^®^ system was housed within a large barn with direct access to animal and anesthesiology facilities, with the study site located at ~67 m altitude (absolute atmospheric pressure at ~101 kPa).

The decompression chamber was modified to allow for several additional sealable ports to be placed, allowing for additional cabling to be run through and power equipment within the chamber, without compromising vacuum pressure. The chamber was lit by two dimmable LED lighting strips (RS PRO White LED Strip, RS Components, UK), set to 180 lux and positioned to the left and right of the central line on the ceiling. Two temperature and relative humidity loggers (Tinytag Ultra 2, TGU-4500, Gemini Data Loggers, UK) were placed at pig level and set to record data at 10 s intervals. To record and monitor both pig behavior and anesthetic monitors (within the chamber), two EzcctvGeoVision surveillance systems (GV1480-16 camera video capture card, ezCCTV, UK) were installed outside the chamber and connected through the sealable ports to multiple CCTV cameras, secured in multiple locations by a custom-built camera rig supplemented with adjustable arms and clamps (Manfrotto, UK). The first system monitored and logged the behavior of each pig with individual cameras from a frontal (facial) angle (Sony GametEffio, SpyCameraCCTV, UK) and aerial cameras (CCD Bird Box Camera, SpyCameraCCTV, UK). The second system involved individual cameras (Bullet LED, SpyCameraCCTV, UK) focused on each anesthetic and BIS monitors within the chamber. The surveillance systems provided not only recorded footage for later in-depth analysis but also live footage of the pigs and the anesthetic monitors to three desktop monitors (Dell, UK) outside of the chamber, allowing for immediate assessment of each pig pre-, during, and post-treatment cycle. Furthermore, two external dynamic microphones (Shure, UK) were fitted inside the chamber at pig height, approximately 50 cm in front of each pig. The microphones were connected to a portable audio recorder (Tascam DR 100-MKII Linear PCM recorder, Tascam, USA).

Based on previous decompression studies in poultry (26, 28–30, 43) and mammals (including humans) (37, 38, 44, 45), we initially selected three target decompression rates to apply to pigs: 60, 80, and 100 ms^−1^ over a total cycle time of 720 s (12 min), which we hypothesized would cause the pigs to enter a non-recovery state with minimal pathological consequences. Crucially, these rates also matched hypothesized feasible cycle times for the commercial slaughter of pigs (25). The study was flexibly designed to allow the inclusion of additional refinement curves, based on preliminary findings as the study progressed. Decompression cycles were applied to all 60 pigs, in pairs, across 6 days in total, with the first 3 days including only the initial pre-selected decompression rates. Following this, preliminary analyses were conducted (three cycle pairs of pigs per treatment), and subsequently two additional “refinement” curves were added to the experiment, which included a slower rate of 40 ms^−1^ (cycle time = 720 s) and a matched rate of 60 ms^−1^, but with a reduced cycle time of 480 s, to examine whether a reduced “hold” period at low pressure was related to pathological outcomes.

### Experimental procedure

Male and female pigs were randomly assigned into mixed pairs, blocked by home pen, to ensure familiarity and reduce the stress associated with individual housing (46). Pairs were initially randomly assigned to one of three decompression treatments according to a randomized-block factorial design using a Latin square. The assignment was blocked by a home pen (to prevent distress with single pairs of pigs being left overnight in home pens) (46). However, following the inclusion of the additional two refinement decompression treatments, the remaining pigs (42 pigs) were reassigned according to a second randomized-block factorial design using a Latin square to one of five decompression treatments, blocked by home pen and partially by day. The experiment took place over 6 days, with 6 pigs (3 cycles) being exposed for the first 2 days and 12 pigs (6 cycles) exposed per day for the remaining 4 days. As a result, a total of 12 pigs (6 pairs) were exposed to one of five decompression treatments.

**Figure 1:**
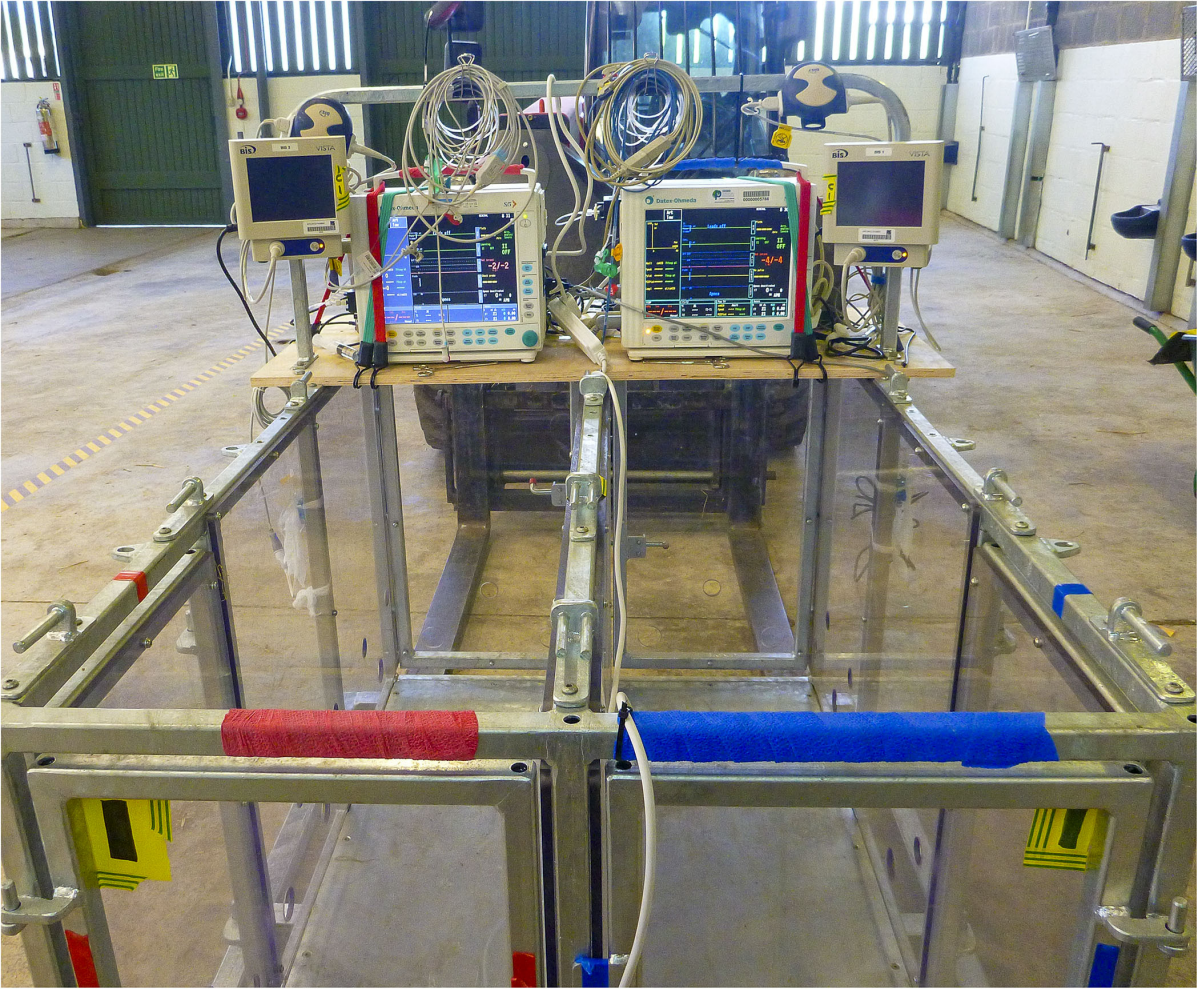
Custom-built holding crate consisting of a galvanized steel frame with clear polycarbonate sides and doors with top tray housing physiological monitors. The crate allowed pairs of pigs to be safely housed in slings in individual compartments during terminal treatments with an unobstructed 360° view of the animals from the CCTV cameras. The base frame of the crate was manufactured to allow maneuverability *via* forklift truck.

Following the completion of anesthesia monitoring instrumentation, each anesthetized pig was placed in an adapted dog surgical sling, designed with four leg openings. The pig (within the sling) was then carefully lifted into a handling crate (L:1500xW:1007xH:800 cm) equipped with struts, allowing the pig within the sling to be suspended in an upright position. The crate was custom-built and consisted of a galvanized steel frame with clear polycarbonate sides and doors—enabling pairs of pigs to be safely housed in individual compartments during stunning with an unobstructed view of the closed-circuit television (CCTV) cameras within the chamber. A shelf was fitted to the back of the crate and housed the anesthesia and BIS monitors (Figure 1).

Once pairs of anesthetized pigs were placed in the crate, it was immediately maneuvered *via* forklift truck to the decompression chamber and carefully positioned to ensure clear camera views of the pigs and the monitors. Individual pig propofol infusion was maintained during transport *via* fluid lines. During decompression, these were passed through modified sealable ports of the chamber and ran through installed ceiling rails, which allowed infusion pump control from outside the chamber, but without compromising the internal vacuum. At all times during the decompression cycle, pigs were continuously administered propofol intravenously at 0.2 mgkg^−1^minute^−1^. A lethal dose of pentobarbital (80 mgkg^−1^) was connected to the fluid lines *via* a three-way stopcock for use if immediate euthanasia became necessary.

The chamber door was then closed, and a 30-s baseline recording for physiological and behavioral measures commenced. The decompression treatment was then applied, according to design allocation. During the cycle, the live footage of the anesthetic monitors was constantly observed by a veterinary anesthetist and a senior scientist who monitored anesthetic depth and confirmed timings of cardiac arrest (based on loss of pulse), cessation of breathing and brain death (as indicated by BIS) in real-time. Trained staff provided real-time monitoring of decompression cycle parameters as indicated by the PLC output. Following confirmation of cardiac arrest and brain death, the cycle was terminated at the prescribed cycle length (e.g., 720 or 480s, according to treatment) and the chamber was immediately recompressed at a fixed rate over 60 s and the door was opened. The crate was removed *via* forklift and death was confirmed by a veterinarian based on the absence of heart sounds (on auscultation) and cranial nerve reflexes. The pigs were removed from the crate and the sensors, loggers, and intravenous cannula were removed.

### Reflexive behavior monitoring

The behavior of each pig was video recorded from a frontal (facial) angle (Sony GametEffio, SpyCameraCCTV, UK) and aerial cameras above each pig (CCD Bird Box Camera, SpyCameraCCTV, UK) using a GeoVision surveillance system (GV1480, ezCCTV, UK). Behavioral footage was analyzed by a trained observer using Noldus Observer XT12 (https://www.noldus.com) using the ethogram in Table 1, blinded to decompression treatment. The ethogram was devised to focus on measures indicative of hypoxia and death in anesthetized animals suspended in modified slings. Behavioral data consisting of latencies, durations, and counts were exported from the Observer to Microsoft Excel.

**Table 1:** Ethogram.

**Behavior**	**Description [including modifier(s)]**	**Measure**
Normal breathing	Regular rhythmic movement of the dorsal thoracic/diaphragm area causing little or no movement in the suspended animal	State (latency, duration)
Abnormal breathing (hyperventilation)	Irregular, non-rhythmic, deep, erratic movement of the thoracic/diaphragm area causing irregular larger movement in the suspended animal	State (latency, duration)
Tachypnoea	Regular, rapid, shallow movement of the thoracic/diaphragm area causing regular repetitive but modest movement of the suspended animal	State (latency, duration)
Cessation of breathing	No movement of the dorsal thoracic/diaphragm area indicative of permanent cessation of breathing	State (latency)
Ear flick	A quick, sudden movement of the ear independent of other body movements but with minimal magnitude or duration	Point event (latency, counts)
Head shake	Quick, sudden movements left and right (side to side) isolated to the head	Point event (latency, counts)
Whole body movements	Sudden, non-regular, spasmodic movement of the whole body which causes up and down head movement and can incorporate non-independent leg movement due to the animal being suspended. Further classified based on magnitude: single (lasts < 1s); repeated (>2 with no gap between events); and prolonged (single event of >1s).	Point event (latency, counts)
Leg movements	Movement of the limb (modifier 1: front left, front right, back left and back right) to a certain level of magnitude (modifier 2: flick, twitch, contract and paddle) separately from movement caused by any of the behavior above	Point event (latency, counts)
Nasal Discharge	Fluid exiting the nostrils throughout the procedure. Logged behavior for each new discharge event seen. Further classified based on type of discharge: clear fluid; bubbles only; and blood.	Point event (latency, counts)
Loss of jaw tone	Lower mandible relaxes and skeletal muscles become more relaxed and lower jaw declines a small distance if suspension in the harness allows	Point event (latency)
Abdominal swelling	Abdominal area begins to expand of the suspended animal	Point event (latency)

### Statistical analyses and data processing

ECG data from the loggers were continuously recorded at a sampling rate of 1,000 Hz, downloaded as text files, and exported to LabChart 8 (ADInstruments, Australia). Heart rate (HR) peaks were automatically detected and ectopic beats were removed as well as band-pass filtering (0.1–48 Hz) was applied to the raw waveforms. All waveforms and HR peaks were then manually checked. HR and heart rate variability (HRV) were sampled every 5 s from clean (artifact-free) waveform excerpts during the 30 s baseline period and throughout the decompression treatment cycles. HRV was determined by automated detection of RR intervals (the time interval between successive R waves on the electrocardiogram) within 5 s clean epochs using the HRV module (2.3) (ADInstruments, Australia).

Statistical analysis was performed using R software version 4.1.3 (R 47) through R Studio (2022.02.1 Build 461, RStudio, PBC, 2009-2022). Data were processed and tidied using the tidyverse package (47). The first comparisons involved modeling for differences between rates with a total cycle time of 720 s (40, 60, 80, and 100 ms^−1^). Secondary comparisons explored differences between cycle lengths within the 60 ms^−1^ decompression rate only, where within-models cycle rate was replaced with cycle length as a fixed effect.

Time series linear mixed models (LMMs) were used to assess changes in physiological parameters (e.g., HR, HRV, BIS, and SpO_2_) over time during each cycle (encompassing baseline, decompression, and hold phase) using the lme and corARMA functions from the nlme package (48, 49), providing an autocorrelation-moving average correlation structure of order for time. All minimal models included ambient temperature, relative humidity, pig weight, and time as covariates, and fixed effects included cycle decompression rate, sex, and crate side, and all interactions between them. Pig ID nested within Pair was included as a random effect. Model fitness was confirmed using the DHARMa package (50), and the residuals of all models were in accordance with uniformity assumptions.

Latency to cardiac arrest was defined by permanent loss of mechanical cardiac activity, represented by the time at which the pulse volume contour on the pulse plethysmograph became permanently imperceptible. Comparisons of latencies to this point event were assessed with generalized linear mixed models (GLMMs) [glmmTMB (51)]. All minimal models included ambient temperature, relative humidity, baseline HR mean, BIS at the point of cardiac arrest, and pig weight as covariates, and fixed effects included cycle decompression rate, sex, and crate side, and all interactions between them. The family link function was set to Poisson distribution. Pair was included as a random effect. Model fitness was confirmed using the DHARMa package (50), and the residuals of all models were in accordance with uniformity assumptions.

Behavioral data comparisons (including latencies, durations, and counts) were evaluated with either GLMMs [glmmTMB (51)] or LMMs [lme4 (52)]. All minimal models included ambient temperature, relative humidity, and pig weight as covariates, and fixed effects included cycle decompression rate, sex, and crate side, and all interactions between them. Pair was included as a random effect. Model fit was determined by examination of residuals *via* the DHARMa package (50). For GLMMs, the family link function was set to either negative binomial distribution with a quadratic parameterization (nbinom2) or Poisson distribution, dependent on model fit and overdispersion parameters (53).

We assessed the significance of explanatory variables for all models with the ANOVA function in the car package (54), with statistical significance based on a *p* < 0.05 threshold, and to identify differences between fixed effects and interactions. Estimated pairwise comparisons were derived with the emmeans package (55), using Tukey adjustment of the *p-*values to account for multiplicity. Where appropriate, the same package was used to assess linear trends between covariates and fixed effects. Graphical summaries were produced using the corrected pairwise comparisons using the ggplot2 package (56) with both standard error (SE) and 95% confidence intervals (CIs) reported. Unless stated, we found no effects of sex, crate side, or co-variates such as weight or environmental parameters.

## Results

**Figure 2:**
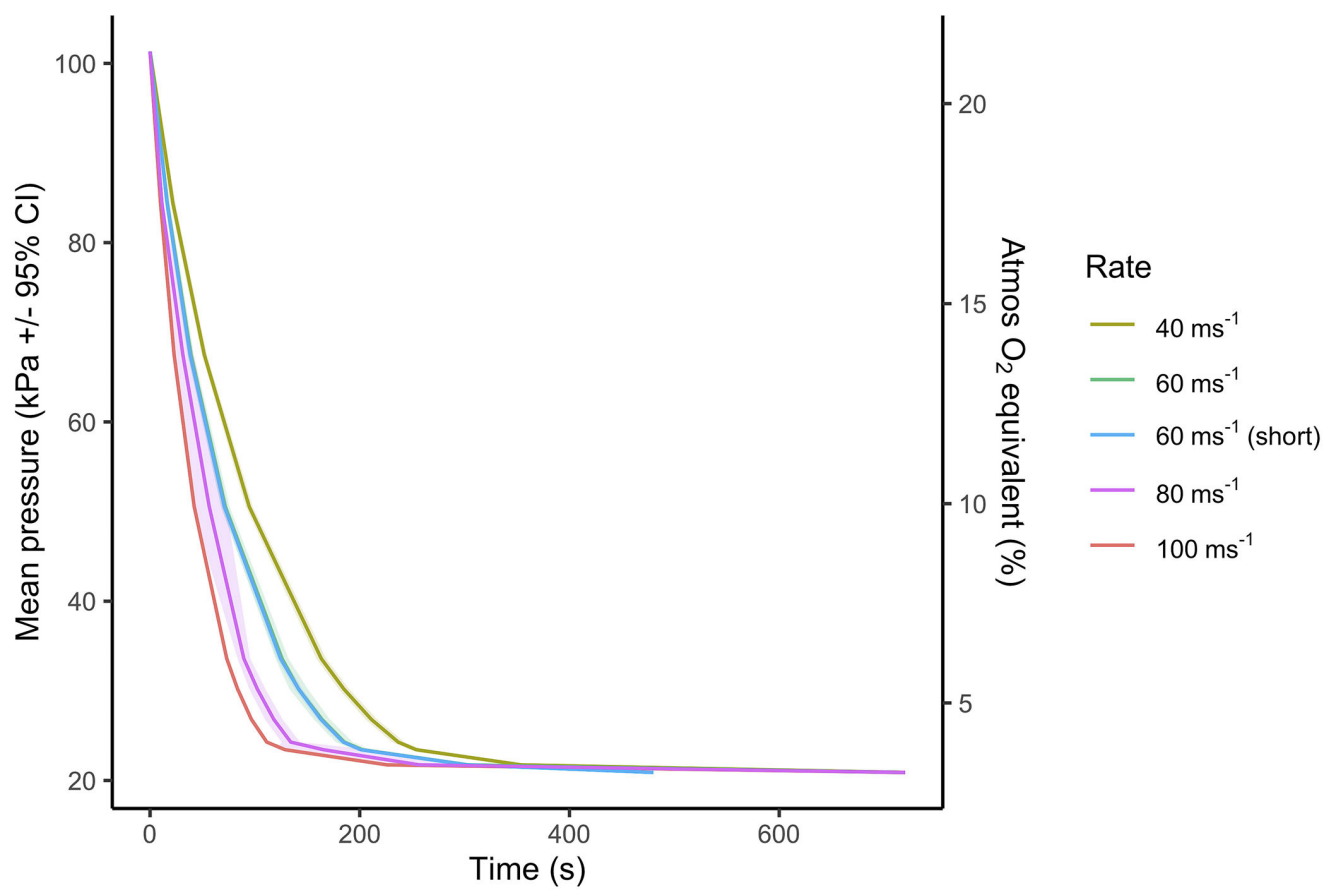
Mean ± 95% CIs pressure (kPa) over time (s) of the five decompression treatment profiles. Each decompression treatment involved a two-phase process, where in the initial phase the chamber was gradually decompressed according to treatment average rate (40, 60, 80, or 100 ms^−1^) to a target pressure of 33 kPa (~8,459 m equivalent altitude) in ~163, ~125, ~90, and ~73 s, respectively. Following target pressure being reached, the second phase (hold phase) involved slowing the decompression to the final absolute vacuum of ~20 kPa (equivalent to 11,498 m) and maintaining the absolute vacuum to the two fixed cycle lengths of 720 s (12 min) for all decompression rates and an additional shorter cycle of 480 s (8 min) for the 60 ms^−1^ rate only. A scaled indication of comparable atmospheric oxygen (O_2_) equivalent as a percentage (%) of inspired air at sea level, using a saturated water vapor value of 6.3 kPa is displayed on the secondary y-axis.

All decompression cycles, irrespective of rate or length, resulted in the death of the pigs, and none required emergency euthanasia. Exact target decompression cycle rate averages were challenging to accomplish due to changes in ambient temperature and relative humidity within the outdoor shed throughout the day, resulting in actual decompression rates achieved being overall lower than desired, especially for the faster decompression rates (Table 2; Figure 2). However, within treatment categories, the rates achieved were consistent. At the start of the cycles, ambient temperature [mean (±SE) = 14.1 ± 0.4°C, mi*n* = 9.8°C, max = 19.5°C] and humidity [mean (±SE) = 66.1 ± 1.2%, mi*n* = 48.9%, max = 81.5%] did not significantly differ between treatments. During all cycles, a percentage reduction from the baseline ~-20% within the chamber was seen for both relative humidity and ambient temperature. Most of these changes occurred within the first 150 s of a decompression cycle and then marginally recovered during the hold phase (Supplementary Figure S1).

**Table 2:** Summary of hypobaric hypoxia treatments, including target decompression rate, N of pigs per treatment, cycle length, achieved decompression rates, and percentage changes of temperature and relative humidity within the chamber during a decompression cycle.

**Target decompression rate (ms^−1^)**	***N***	**Cycle length (s)**	**Mean (95% CI) decompression rate (ms^−1^)**	**Mean (95% CI) percentage change in relative humidity (%)**	**Mean (95% CI) percentage change in temperature (%)**
40	12	720	42.27 (41.19, 43.36)	−19.23 (−22.62, −17.27)	−17.65 (−21.44, −13.85)
60	12	720	54.09 (51.66, 56.52)	−22.54 (−25.32, −19.76)	−21.09 (−24.93, −17.26)
60	12	480	54.27 (53.06, 55.48)	−21.36 (−24.07, −18.65)	−22.50 (−26.55, −18.45)
80	12	720	74.75 (70.31, 79.19)	−21.68 (−24.94, −18.43)	−18.86 (−22.53, −15.19)
100	12	720	89.84 (88.45, 91.23)	−23.18 (−26.55, −19.81)	−17.17 (−20.64, −13.70)

There were two unexpected incidents during two decompression cycles, both caused by a malfunction with the anesthesia equipment (syringe driver malfunction) external to the chamber, which resulted in temporary aspiration of air and rapid administration of propofol to three pigs [cycle run 16 (80 ms^−1^), both pigs and cycle run 28 (80 ms^−1^), a single pig]. These occurred late in the cycle, therefore the physiology and behavior data up to the incidents were included but these animals were excluded for pathology [reported in (41)].

### Physiological findings

**Figure 3:**
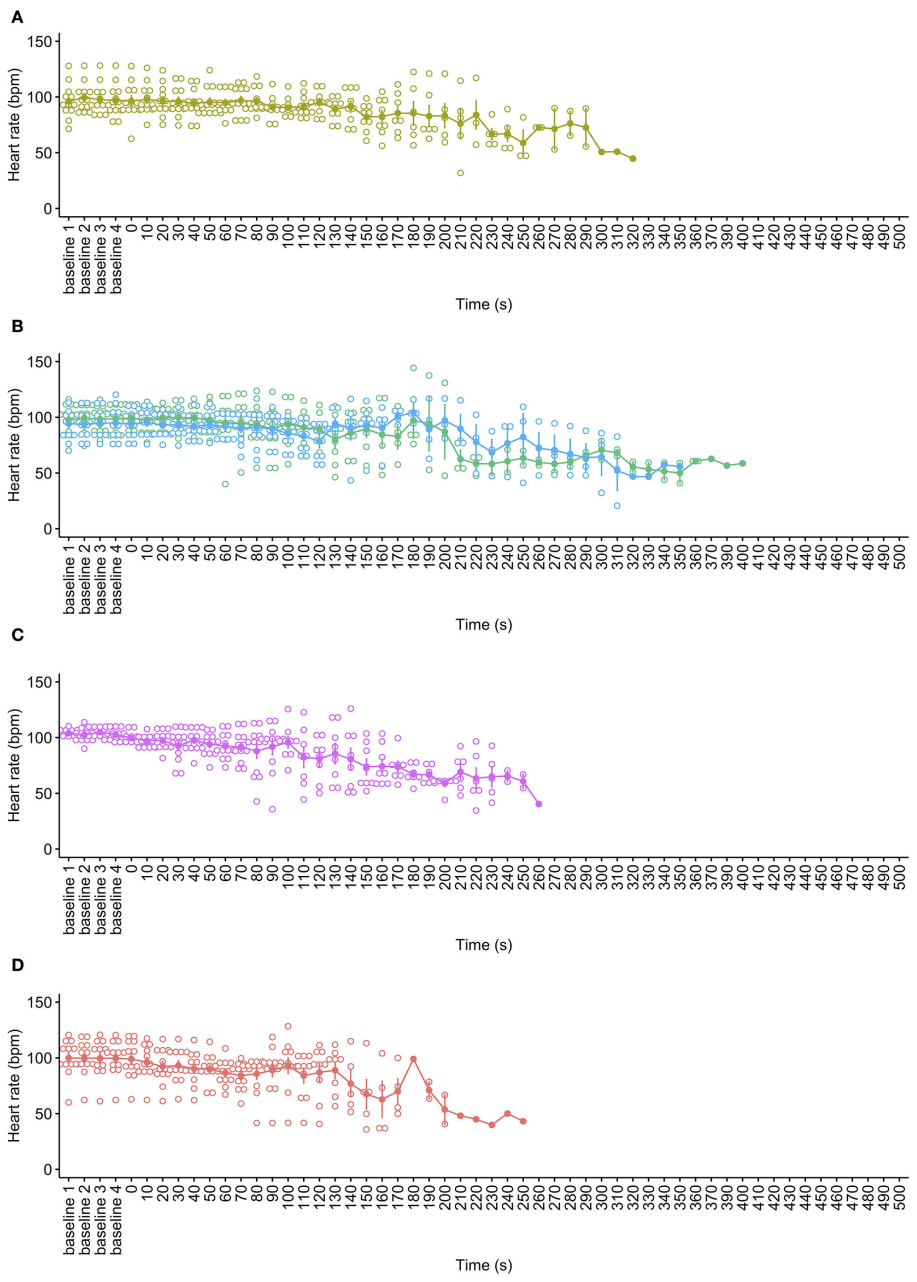
Mean ± SE heart rate (beats per minute) over time for each decompression treatment rate: **(A)** 40 ms^−1^ (yellow); **(B)** 60 ms^−1^ long/720 s cycle (green) and 60 ms^−1^ short/480 s cycle (blue); **(C)** 80 ms^−1^ (pink); and **(D)** 100 ms^−1^ (red). Mean is represented by solid dots and with error bars for SE, with hollow dots representing individual raw data points. The x-axis has been limited to the baseline period and 500 s into the decompression cycles only. Individual HR data were omitted post cardiac arrest, defined by permanent loss of mechanical cardiac activity, represented by the time at which the pulse volume contour on the pulse plethysmograph became permanently imperceptible.

There was no difference in mean baseline heart rates across all pigs for all cycle rate treatments [$X_{\left(1,28\right)}^{2}$ = 3.167, *p* = 0.367]. All decompression treatments were associated with progressive declines in HR (hypoxic bradycardia) over time, as expected [$X_{\left(1,58\right)}^{2}$ = 323.35, *p* < 0.0001, Figure 3]. Time series modeling detected no overall effect of cycle rate [$X_{\left(3,58\right)}^{2}$ = 5.02, *p* = 0.170] on HR or the interaction between time and cycle rate [$X_{\left(3,58\right)}^{2}$ = 5.92, *p* = 0.115], with all decompression rates demonstrating consistent trends of gradual decreases in HR per 5 s time interval (mean trends: 40 ms^−1^ = −0.06 bpm (95% CI −0.07,−0.04); 60 ms^−1^ = −0.06 bpm (95% CI −0.07,−0.04); 80 ms^−1^ = −0.08 bpm (95% CI −0.09,−0.06); and 100 ms^−1^ = −0.06 bpm (95% CI −0.08,−0.05). Overall, female pigs had higher HR than male pigs during the decompression cycles [$X_{\left(1,58\right)}^{2}$ = 8.40, *p* = 0.004], but there was no difference in HR trends over time by sex (mean trends: male = −0.06 (95% CI−0.07, −0.05); female = −0.07 (95% CI −0.08, −0.06), t_ratio_ = 0.967, *p* = 0.334). Additionally, there was no difference in trends over time between the sexes within decompression cycle rates. There were no notable increases in HR during decompression. Some electrical activity (as captured by ECG electrodes but not resembling QRS complexes) commonly persisted for a long period after cardiac and respiratory arrest and was present on exit from the chamber in some animals.

HRV analysis revealed a general trend of RR interval increase as cycles progressed, with no differences overall in HRV based on cycle rate [$X_{\left(3,58\right)}^{2}$ = 4.62, *p* = 0.202, Supplementary Figure S2]. However, there was an effect on the interaction of cycle rate and time [$X_{\left(3,58\right)}^{2}$ = 12.79, *p* = 0.005], with pairwise comparisons revealing the 80 ms^−1^ cycle rate resulting in greater increases in HRV over time [trend: 0.001 (95% CI 0.001, 0.001)] compared to both the 40 ms^−1^ [trend: 0.000 (95% CI 0.000, 0.001), t_ratio_ = −2.64, *p* = 0.041] and 60 ms^−1^ [trend: 0.000 (95% CI 0.000, 0.001), t_ratio_ = −3.20, *p* = 0.008], but not different to the 100 ms^−1^ cycle rate [trend: 0.001 (95% CI 0.001, 0.001), t_ratio_ = 1.94, *p* = 0.211], and no pairwise differences between 40 ms^−1^, 60 ms^−1^, and 100 ms^−1^. Overall, male pigs had higher HRV compared to female pigs [$X_{\left(1,58\right)}^{2}$ = 7.11, *p* = 0.008]; however, there was no effect when interacting within cycle rate [$X_{\left(3,58\right)}^{2}$ = 5.72, *p* = 0.126] and over time during the cycle [$X_{\left(4,58\right)}^{2}$ = 9.23, *p* = 0.056].

**Figure 4:**
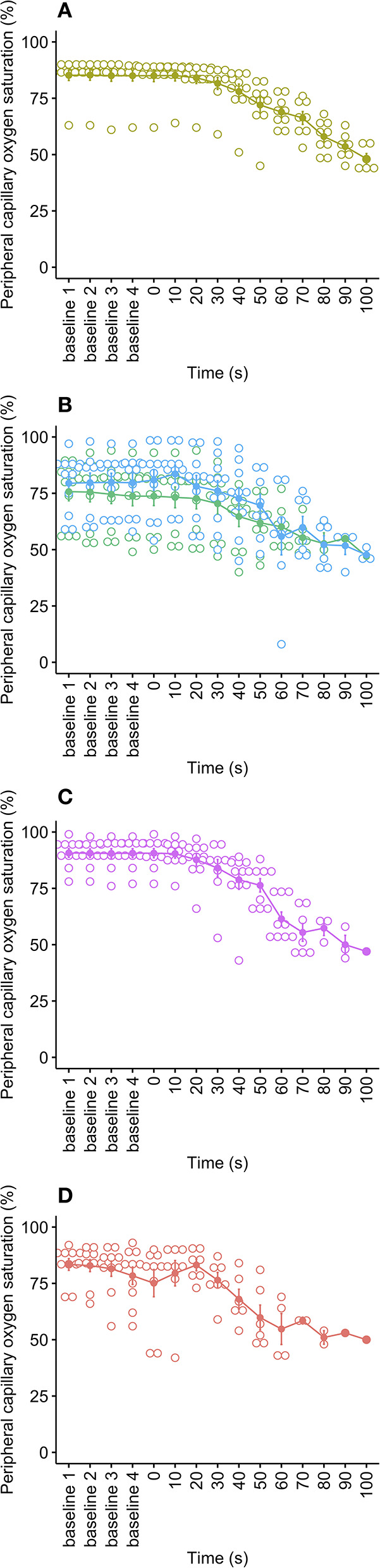
Mean ± SE peripheral capillary oxygen saturation (SpO_2_, %) over time for each decompression treatment rate: **(A)** 40 ms^−1^ (yellow); **(B)** 60 ms^−1^ long/720 s cycle (green) and 60 ms^−1^ short/480 s cycle (blue); **(C)** 80 ms^−1^ (pink); and **(D)** 100 ms^−1^ (red). Mean is represented by solid dots, with error bars for SE, and with hollow dots representing individual raw data points. Due to SpO_2_ data becoming increasingly unreliable and sparse as the cycles progressed, the plots have been limited to the baseline period and the first 100 s of the cycle.

Peripheral capillary oxygen saturation (SpO_2_) is an estimate of the amount of oxygen in the blood and was displayed as a numerical percentage value from each anesthesia monitor. The mean oxygen saturation during baseline for all pigs was 82.3 ± 0.8% (range 49–99%), reflecting that the anesthetized pigs were often already hypoxic, with ranges due to the variation in anesthetic depths achieved, but no effect of cycle rate on mean baseline values [$X_{\left(1,28\right)}^{2}$ = 4.84, *p* = 0.184]. This is a result of maintenance under general anesthesia (which depresses respiration) without supplementary oxygen. As the decompression cycles progressed, SpO_2_ data became increasingly unreliable and sparse as the sensors are designed to work within normal physiological limits and normal ambient atmospheric pressures. As a result, data were limited within the first 60 to 100 s of decompression cycles, with low N for each data point (1–7 pigs per cycle rate), making time series modeling unreliable. Based on graphical observations (Figure 4), the faster decompression rates (80 and 100 ms^−1^) resulted in sharper reductions in SpO_2_ (from around 20 to 30 s into the cycle) compared to the slower rates (40 and 60 ms^−1^).

**Figure 5:**
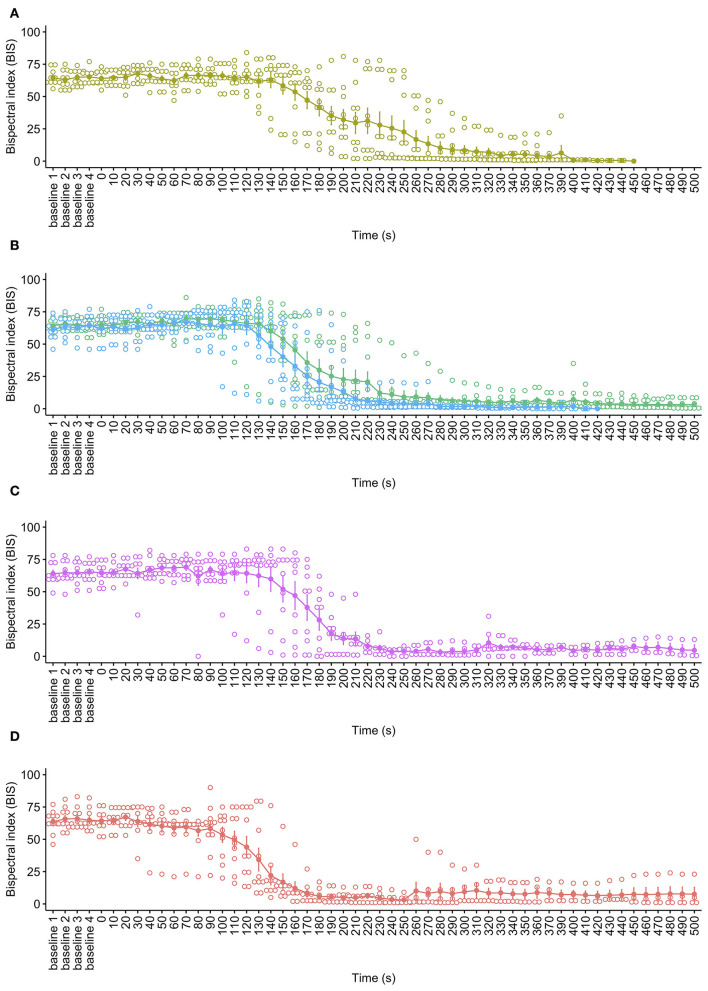
Mean ± SE bispectral index (BIS) over time for each decompression treatment rate: **(A)** 40 ms^−1^ (yellow); **(B)** 60 ms^−1^ long/720 s cycle (green) and 60 ms^−1^ short/480 s cycle (blue); **(C)** 80 ms^−1^ (pink); and **(D)** 100 ms^−1^ (red). Mean is represented by solid dots with error bars for SE, with hollow dots representing individual raw data points. Due to BIS data becoming increasingly unreliable and sparse as the cycles progressed [e.g., reaching EEG silence (< 10)], the plots have been limited to the baseline period and the first 500 s of the cycle.

BIS is used to monitor the depth of anesthesia using a statistically based, proprietary calculation involving the weighted sum of several electroencephalographic parameters. The BIS monitor provided a single number, ranging from 0 (equivalent to EEG silence) to 100 (fully conscious), based on a rolling average every 2 s. At baseline, the mean BIS value ranged from 64.4 ± 0.5 reflecting expected values due to general anesthesia. While there was some variation in anesthetic depth, there was no effect of cycle rate on mean baseline BIS values [$X_{\left(1,28\right)}^{2}$ = 1.11, *p* = 0.774]. Observations of BIS values showed a pattern of a marginal initial increase for the 40, 60, and 80 ms^−1^ cycle rates in the first 100 s of the cycles; however, these were not statistically significant increases from baseline periods (Figure 5). The pigs undergoing the 100 ms^−1^ cycle rate showed no such increase. Following this period all pigs, irrespective of cycle rate showed a sharp decline in BIS values, the timing of which was related to decompression treatment. At 40, 60, and 80 ms^−1^, the sharp BIS decline began between 120 and 160 s into the cycle, while for 100 ms^−1^, this occurred earlier, at around 60 to 90 s, and the drop was steeper across the whole cycle. However, there was no effect of cycle rate for overall BIS values [$X_{\left(3,58\right)}^{2}$ = 1.68, *p* = 0.644] or as an interaction with time [$X_{\left(3,58\right)}^{2}$ = 3.17, *p* = 0.366]. Declining BIS reflected reduced brain function as hypoxia progressed, and very low BIS values (e.g., <10) reflect brain death. As with SpO_2_, however, the accuracy of BIS measures out with normal physiological values, and normal ambient atmospheric pressures are limited, so an accurate time to brain death could not be determined, and this is highlighted by the marked variation in the data as atmospheric pressure dropped and brain function reduced.

**Figure 6:**
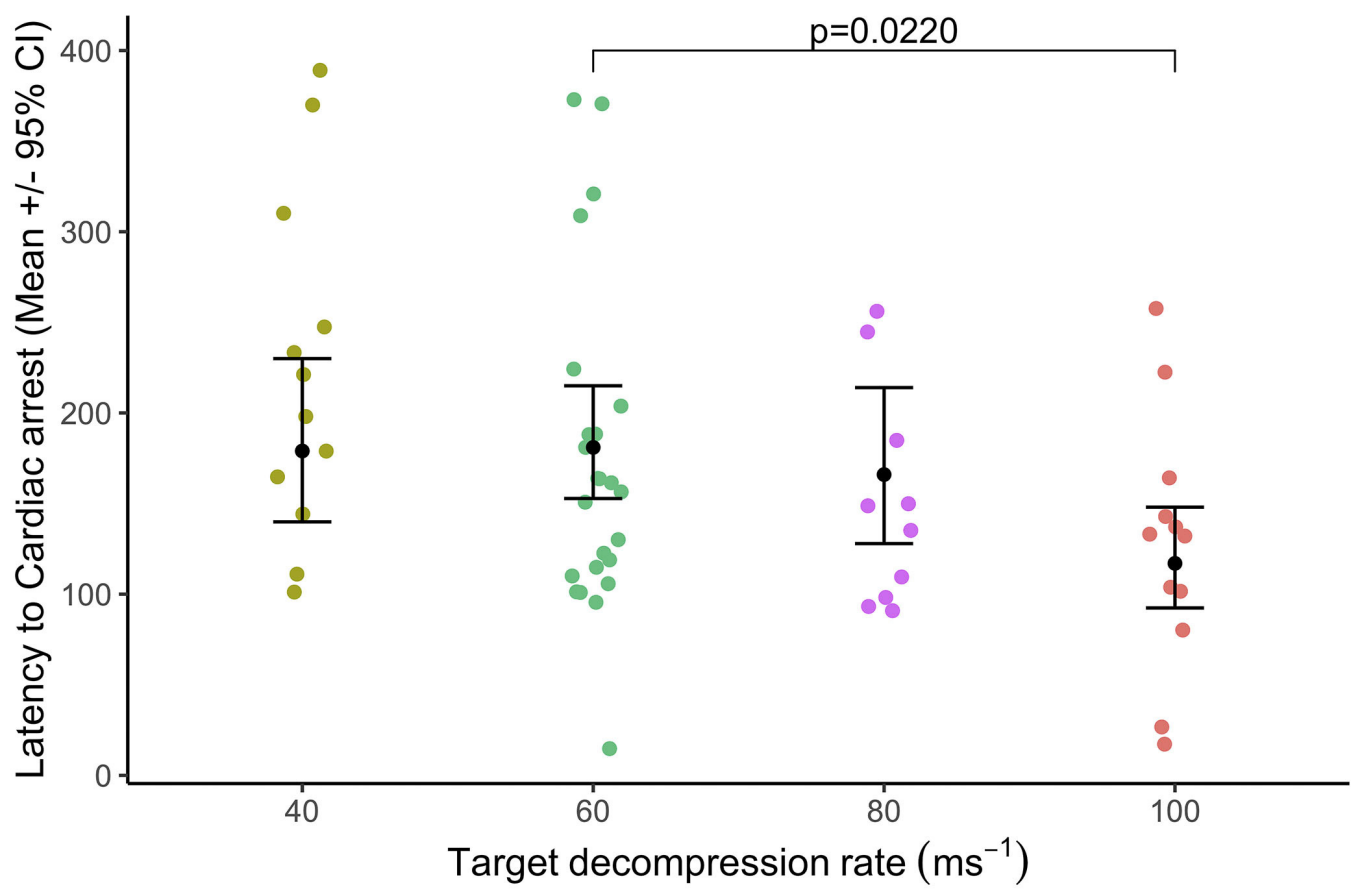
Mean (±95% CIs) latency to cardiac arrest relative to target decompression cycle rates: 40 ms^−1^ (*n* = 12), 60 ms^−1^ (*n* = 24), 80 ms^−1^ (*n* = 10), or 100 ms^−1^ (*n* = 12). Cardiac arrest was defined by permanent loss of mechanical cardiac activity, represented by the time at which the pulse volume contour on the pulse plethysmograph became permanently imperceptible. Significant pairwise comparisons are annotated only.

**Figure 7:**
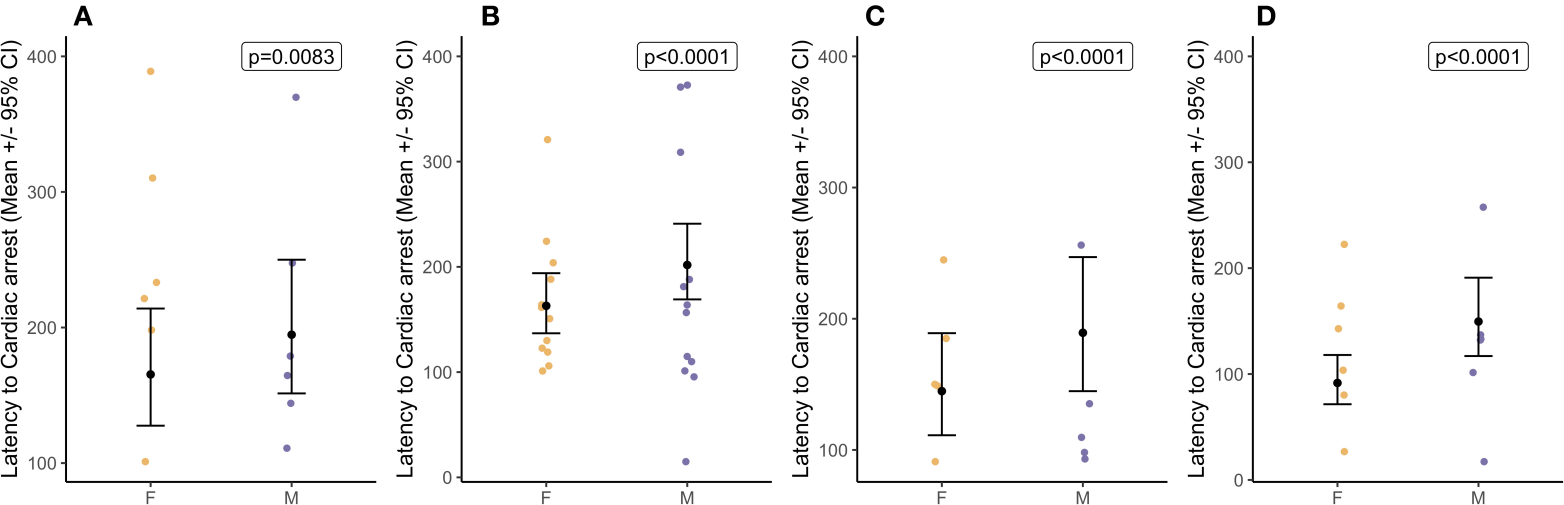
Sex differences (mean ± 95% CIs) in latency to cardiac arrest for each target decompression cycle rates: **(A)** 40 ms^−1^ (*n* = 12), **(B)** 60 ms^−1^ (*n* = 24), **(C)** 80 ms^−1^ (*n* = 10), and **(D)** 100 ms^−1^ (*n* = 12).

Latency to cardiac arrest was defined by permanent loss of mechanical cardiac activity, represented by the time at which the pulse volume contour on the pulse plethysmograph became permanently imperceptible. Decompression rate had an overall effect [$X_{\left(3,58\right)}^{2}$ = 9.79, *p* = 0.020] on latency to cardiac arrest which was from 120 to 183 s [40 ms^−1^ = 178.0 ± 22.2 s; 60 ms^−1^ = 183.0 ± 15.7 s; 80 ms^−1^ = 163.0 ± 21.1 s; and 100 ms^−1^ = 120.0 ± 14.2 s (Figure 6)], and there was no effect of baseline heart rate ranges [$X_{\left(1,58\right)}^{2}$ = 0.98, *p* = 0.323]. Pairwise comparisons highlighted the only difference between 60 and 100 ms^−1^ (t_ratio_ = 3.04, *p* = 0.022) with cardiac arrests occurring earlier during the faster 100 ms^−1^ decompression rate compared to the 60 ms^−1^, but no other pairwise differences. At the point of cardiac arrest, the BIS values ranged between 36 and 58, with BIS values being lower when the cardiac arrest was delayed (Coefficient = −0.01 ± 0.00 (CI = −0.01, −0.01), *p* < 0.0001). Overall, male pigs (183.0 ± 10.5s) showed delayed latencies to cardiac arrest compared to female pigs (138.0 ± 8.0s) [$X_{\left(1,58\right)}^{2}$ = 93.50, *p* < 0.0001], and comparisons between the sexes within the cycle rate showed a similar pattern (Figure 7). Importantly, when the data were a subset to the cycle rate of 60 ms^−1^ only and to conduct comparisons related to cycle length (480 s or 720 s), no differences in latency to cardiac arrest were found [$X_{\left(1,19\right)}^{2}$ = 90.05, *p* = 0.816]. There was an effect of the interaction between cycle length and sex [$X_{\left(1,19\right)}^{2}$ = 40.33, *p* < 0.0001]; however, pairwise comparisons highlighted no differences between the sexes in the shorter cycle (males = 248.0 ± 34.8; females = 228.0 ± 33.8; *t*_ratio_ = −1.46, *p* = 0.178), while in the longer cycle, male pigs showed on average a delayed latency to cardiac arrest compared to female pigs (males = 258.0 ± 15.5; females = 115.0 ± 332.3; *t*_ratio_ = −9.11, *p* < 0.0001). Additionally, pigs with higher baseline heart rates were shown to have delayed latencies to cardiac arrest, irrespective of cycle length (Coefficient = 0.02 ± 0.00 (CI = 0.03, 0.02), *p* < 0.0001).

### Reflexive behavioral findings

Given that the pigs were anesthetized, behavioral output was limited to reflexive and hypoxia-induced changes and no welfare impacts can be determined. Summaries of durations, latencies, and counts of behaviors are shown in Tables 3, 4. All pigs were observed to display normal rhythmic breathing during baseline periods and at the start of decompression cycle treatments. The duration of normal breathing did not vary depending on the cycle rate [$X_{\left(1,28\right)}^{2}$ = 1.80, *p* = 0.615: range 32-60s] nor cycle length within the 60 ms^−1^ rate [$X_{\left(1,12\right)}^{2}$ = 0.44, *p* = 0.5092]. Following the period of normal breathing, between 30 and 67% of pigs (40 ms^−1^ = 6/12 pigs; 60 ms^−1^ = 7/24 pigs; 80 ms^−1^ = 8/12 pigs; and 100 ms^−1^ = 5/12 pigs) shifted into bouts of rapid regular breathing (tachypnoea), but there was no difference in latency to onset of tachypnoea based on cycle rate [$X_{\left(3,26\right)}^{2}$ = 3.22, *p* = 0.358], which ranged between 16 and 49 s, but it should be noted that greater variation was observed in the slowest cycle rate. There was also no effect of cycle rate on the duration of tachypnoea [$X_{\left(3,26\right)}^{2}$ = 2.25, *p* = 0.523], with consistent variation seen within each cycle rate (mean range: 69–100 s). There was no effect of cycle length (within 60 ms^−1^ rate only: 480 s or 720 s) on the latency [$X_{\left(1,7\right)}^{2}$ = 1.99, *p* = 0.1583] or duration of tachypnoea [$X_{\left(1,7\right)}^{2}$ = 1.35, *p* = 0.2446].

**Table 3:** Comparisons of decompression cycle rate (40ms^−1^, 60ms^−1^, 80ms^−1^, and 100ms^−1^) for behaviors observed, including latencies (s) and durations (s) of behaviors performed by an individual pig.

**Behavior**	**Measure**	**Target cycle rate (ms^−1^)**	**Mean±SE**	**95% confidence interval**	***P*** **-value**
Normal breathing	Duration (s)	40	32.0 ± 13.4	13.1, 78.0	0.615 (ns)
		60	59.7 ± 16.6	33.1, 108.0	
		80	34.1 ± 17.6	11.3, 103.0	
		100	42.0 ± 18.5	16.5, 107.0	
Tachypnoea	Latency (s)	40	49.1 ± 23.7	17.8, 135.4	0.358 (ns)
		60	19.2 ± 7.6	8.3, 44.1	
		80	22.8 ± 10.2	8.9, 58.2	
		100	15.9 ± 7.9	5.6, 44.8	
	Duration (s)	40	99.5 ± 19.9	65.4, 151.0	0.523 (ns)
		60	96.5 ± 18.2	64.9, 143.0	
		80	68.8 ± 12.7	46.7, 101.0	
		100	76.3 ± 16.8	48.0, 121.0	
Abnormal breathing	Latency (s)	40	68.3 ± 24.4	9.8, 127.0	0.806 (ns)
		60	78.1 ± 16.1	42.7, 113.0	
		80	99.1 ± 24.2	47.9, 151.0	
		100	56.7 ± 26.9	14.7, 114.0	
	Duration (s)	40	86.4 ± 25.5	46.1, 161.6	0.235 (ns)
		60	53.9 ± 10.2	36.2, 80.4	
		80	30.5 ± 9.4	15.9, 58.8	
		100	41.0 ± 12.8	21.2, 79.3	
Cessation of breathing	Latency (s)	40	205 ± 12.6	174.9, 234.0	< 0.0001 (^***^)
		60	146 ± 12.1	127.9, 164.0	
		80	131 ± 12.1	105.1, 157.0	
		100	114 ± 12.4	86.3, 141.0	
Whole body movements (combined - first occurrence)	Latency (s)	40	215.0 ± 23.3	161.5, 268.0	0.0463 (^*^)
		60	166.0 ± 14.6	133.9, 197.0	
		80	194.0 ± 21.4	147.7, 240.0	
		100	130.0 ± 20.7	84.2, 176.0	
Whole body movements (combined - last occurrence)	Latency (s)	40	474.0 ± 39.3	389.9, 560.0	0.0052 (^**^)
		60	422.0 ± 22.0	375.0, 469.0	
		80	321.0 ± 32.3	251.0, 391.0	
		100	351.0 ± 32.8	280.0, 422.0	
Leg movements (combined)	Latency (s)	40	126.0 ± 27.1	63.6, 250.0	0.8467 (ns)
		60	125.0 ± 17.7	79.6, 196.0	
		80	121.0 ± 21.0	69.4, 210.0	
		100	107.0 ± 16.1	66.7, 173.0	
Ear flicks	Latency (s)	40	5.4 ± 2.4	2.0, 14.9	0.6090 (ns)
		60	5.5 ± 2.0	2.4, 12.6	
		80	3.5 ± 1.3	1.5, 8.3	
		100	2.5 ± 1.3	0.7, 8.3	
Nasal discharge (combined)	Latency (s)	40	417.0 ± 99.8	249.0, 700.0	0.5064 (ns)
		60	296.0 ± 62.0	189.0, 466.0	
		80	442.0 ± 108.5	260.0, 751.0	
		100	437.0 ± 81.1	293.0, 653.0	
Abdominal swelling	Latency (s)	40	417.0 ± 99.8	249.0, 700.0	0.9807 (ns)
		60	296.0 ± 62.0	189.0, 466.0	
		80	442.0 ± 108.5	260.0, 751.0	
		100	437.0 ± 81.1	293.0, 653.0	

Mean (±SE) and 95% confidence intervals are reported for each behavior. *P*-values are annotated for clarity: ns, not significant;

* *p* < 0.05;

** *p* < 0.01;

and

*** *p* < 0.001.

**Table 4:** Comparisons of target decompression cycle rate (40ms^−1^, 60ms^−1^, 80ms${}_{,}^{-1}$ and 100ms^−1^) for frequencies of behaviors performed by an individual pig.

**Behavior**	**Measure**	**Target cycle rate (ms^−1^)**	**Mean±SE**	**95% confidence interval**	***P*** **-value**
Whole body movement (prolonged)	Frequency	40	1.9 ± 0.6	0.9, 4.0	0.813 (ns)
		60	1.3 ± 0.3	0.8, 2.0	
		80	0.0 ± 0.0	0.0, 4.0	
		100	1.2 ± 0.5	0.5, 3.0	
Whole body movement (repeat)	Frequency	40	3.8 ± 0.9	2.3, 6.3	0.0091 (^**^)
		60	2.1 ± 0.4	1.4, 3.2	
		80	0.4 ± 0.3	0.1, 1.5	
		100	2.5 ± 0.7	1.4, 4.5	
Whole body movement (single)	Frequency	40	8.7 ± 2.0	5.4, 13.9	0.2002 (ns)
		60	5.4 ± 0.9	3.9, 7.6	
		80	4.7 ± 1.2	2.8, 7.9	
		100	4.4 ± 1.1	2.6, 7.5	
Whole body movement (combined)	Frequency	40	14.8 ± 2.6	10.3, 21.4	0.0047 (^**^)
		60	9.0 ± 1.2	6.9, 11.8	
		80	5.2 ± 1.2	3.3, 8.2	
		100	8.3 ± 1.6	5.6, 12.3	
Leg movements (combined)	Frequency	40	0.6 ± 0.4	0.2, 2.3	0.0037 (^**^)
		60	0.2 ± 0.1	0.1, 0.6	
		80	2.0 ± 0.6	1.0, 3.8	
		100	1.0 ± 0.5	0.4, 2.7	
Ear flicks	Frequency	40	1.2 ± 1.5	0.1, 14.7	0.2471 (ns)
		60	0.4 ± 0.4	0.1, 3.1	
		80	4.7 ± 3.9	0.8, 26.7	
		100	3.8 ± 3.6	0.5, 27.3	
Nasal discharge (combined)	Frequency	40	1.1 ± 1.5	0.1, 20.0	0.1848 (ns)
		60	0.2 ± 0.2	0.0, 2.0	
		80	0.0 ± 0.0	0.0, 0.0	
		100	5.1 ± 5.3	0.6, 45.0	

Mean (±SE) and 95% confidence intervals are reported for each behavior. *P*-values are annotated for clarity: ns, not significant; *p < 0.05;

** *p* < 0.01;

and ***p < 0.001.

**Figure 8:**
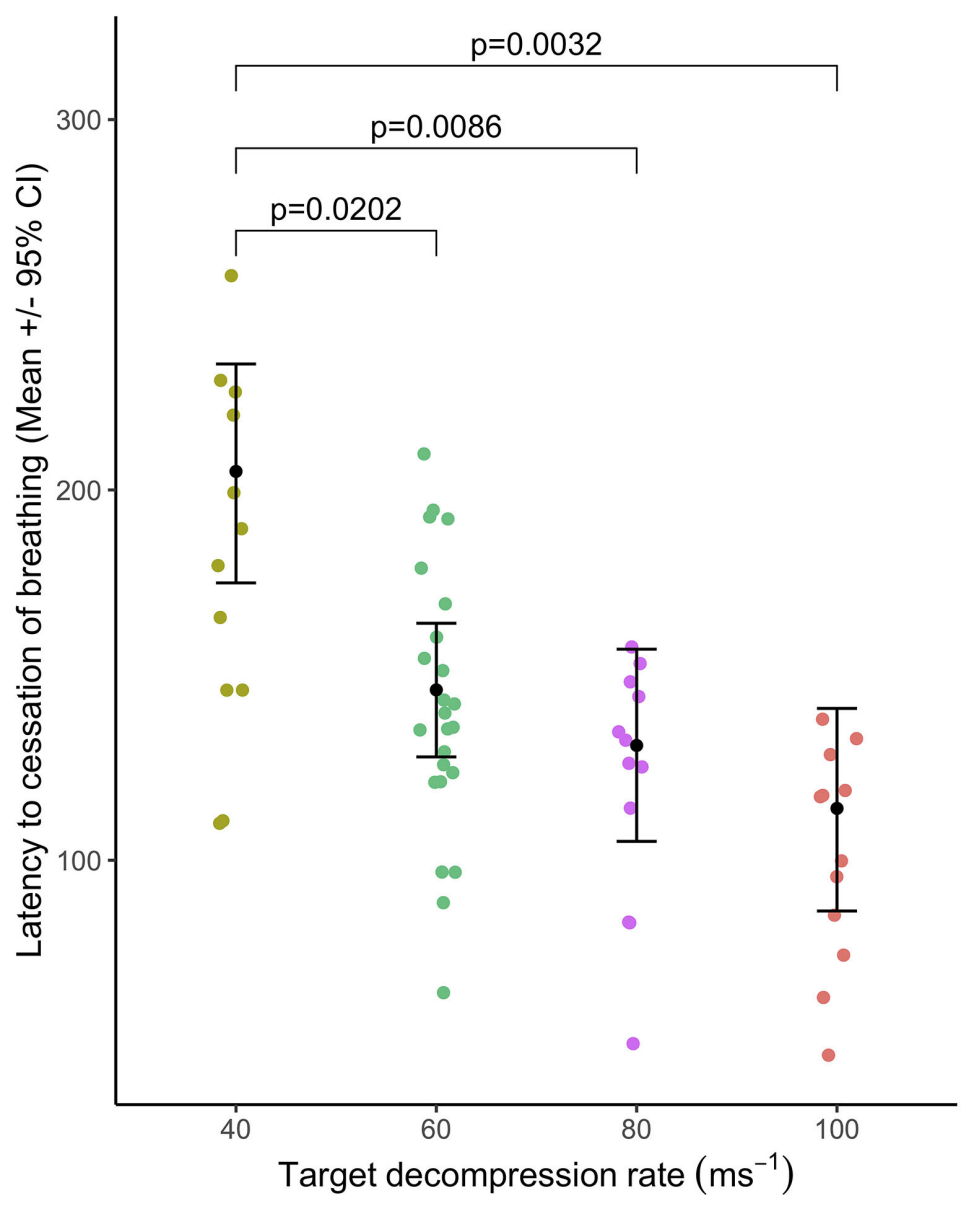
Mean ( ± 95% CIs) latency to cessation of breathing relative to target decompression cycle rates: 40 ms^−1^ (*n* = 12), 60 ms^−1^ (*n* = 24), 80 ms^−1^ (*n* = 12), or 100 ms^−1^ (*n* = 12). Only significant pairwise comparisons are annotated.

The majority of pigs (75–100%) were observed breathing abnormally following either a period of tachypnoea or transitioned directly from normal breathing (40 ms^−1^ = 11/12 pigs; 60 ms^−1^ = 22/24 pigs; 80 ms^−1^ = 12/12 pigs; and 100 ms^−1^ = 9/12 pigs). There was no effect of cycle rate on the latency to abnormal breathing [$X_{\left(3,29\right)}^{2}$ = 0.98, *p* = 0.806: mean ranges 57–99s] nor the duration [$X_{\left(3,29\right)}^{2}$ = 4.26, *p* = 0.235: mean ranges 57–99s]. There was no effect of cycle length (within 60 ms^−1^ rate only: 480 s or 720 s) on the latency [$X_{\left(1,12\right)}^{2}$ = 0.33, *p* = 0.5677]. However, longer durations of abnormal breathing were observed in the longer cycle length (480 s = 29.4 ± 6.8 s and 720 s = 71.0 ± 12.4 s; $X_{\left(1,12\right)}^{2}$ = 6.80, *p* = 0.0091). Latency to the cessation of breathing occurred earlier in the cycle for the faster decompression rates [$X_{\left(3,30\right)}^{2}$ = 28.2, *p* < 0.0001, Figure 8], with pairwise comparisons revealing differences between the slowest rate (40 ms^−1^) and all faster rates (60, 80, and 100 ms^−1^), but no pairwise differences between the 60, 80, and 100 ms^−1^ rates. In total, 19/58 pigs (32.8%) had ceased breathing before cardiac arrest, with cessation of breathing occurring on average 38.2 ± 8.8 s earlier. For pigs where cessation of breathing occurred after cardiac arrest, the average delay of observed breathing ending was 64.3 ± 10.3s. There was no effect of cycle length (within 60 ms^−1^ rate only: 480 s or 720 s) on the latency to the cessation of breathing [$X_{\left(1,12\right)}^{2}$ = 3.60, *p* = 0.0579]. However, male pigs were observed to stop breathing before female pigs (males = 123.0 ± 12.3 s and females = 162.0 ± 13.3 s; $X_{\left(1,12\right)}^{2}$ = 5.67, *p* = 0.0172].

At a similar time to the cessation of breathing, the onset of the first whole-body movements occurred. All pigs except one (exposed to 80 ms^−1^ rate) performed whole-body movements, with the latency to the first occurrence being earlier in the cycle for faster decompression rates [$X_{\left(3,29\right)}^{2}$ = 7.98, *p* = 0.0463]; however, pairwise comparisons failed to identify differences between individual cycle rates. Cycle length (within 60 ms^−1^ rate only: 480 s or 720 s) had no impact on latency to first whole-body movements [$X_{\left(1,12\right)}^{2}$ = 1.80, *p* = 0.1791]. Latency to the last whole-body movements showed the same pattern, with slower rates resulting in the last whole-body movements occurring later in the cycle [$X_{\left(3,29\right)}^{2}$ = 12.77, *p* = 0.0052] compared to faster rates. However, pairwise comparisons only identified a difference between the 40 and 80 ms^−1^ rates (t_ratio_ = 3.02, *p* = 0.044). Only 3.3% of pigs displayed their last whole-body movement before cardiac arrest. Cycle length (within 60 ms^−1^ rate only: 480 s or 720 s) had no impact on latency to first whole-body movements [$X_{\left(1,12\right)}^{2}$ = 0.38, *p* = 0.5363].

Whole-body movements were classified as either prolonged, repeated, or single to differentiate between the severity of the movement (Table 1). For prolonged whole-body movements, more pigs in the slower rates (40 ms^−1^ = 7/12 pigs; 60 ms^−1^ = 13/24 pigs) displayed these behaviors compared to those in the faster rates (80 ms^−1^ = 5/12 pigs; 100 ms^−1^ = 3/12 pigs), but there was no difference in their frequency dependent on cycle rate [$X_{\left(3,30\right)}^{2}$ = 0.95, *p* = 0.813]. Repeated whole-body movements were performed by over half of all pigs irrespective of cycle rate (40 ms^−1^ = 12/12 pigs; 60 ms^−1^ = 17/24 pigs; 80 ms^−1^ = 6/12 pigs; and 100 ms^−1^ = 8/12 pigs), with the frequency of movements dependent on cycle rate [$X_{\left(3,30\right)}^{2}$ = 11.55, *p* = 0.091], indicating higher frequencies in the slower rates. Pairwise comparisons only showed differences between 80 and both 40 ms^−1^ (t_ratio_ = 3.31, *p* = 0.0163) and 60 ms^−1^ (*t*_ratio_ = 2.55, *p* = 0.0814), with the faster rate showing the lowest frequencies. However, there were no pairwise differences between the remaining cycle rate comparisons, including the fastest cycle rate of 100 ms^−1^, therefore, the effect of cycle rate does not appear to directly correlate with the frequency of repeated whole-body movements. Single whole-body movements were performed by the majority of pigs (40 ms^−1^ = 12/12 pigs; 60 ms^−1^ = 24/24 pigs; 80 ms^−1^ = 10/12 pigs; and 100 ms^−1^ = 12/12 pigs) and were also typically the first whole-body movement to be performed. There was no effect of cycle rate on the frequency of single whole-body movements [$X_{\left(3,30\right)}^{2}$ = 4.64, *p* = 0.2002], although numerically, frequencies appeared highest in the slowest cycle rate (40 ms^−1^). When all whole-body movement frequencies (prolonged, repeated, and single) were combined, the slowest cycle rate resulted in the highest number [$X_{\left(3,30\right)}^{2}$ = 12.95, *p* = 0.0047] compared to faster rates, although pairwise comparisons only identified a difference between the 40 ms^−1^ and 80 ms^−1^ (t_ratio_ = 3.58, *p* = 0.0088).

There was no effect of cycle rate on leg movements (combined) [*X*^2^_(3, 13)_ = 0.81, *p* = 0.8467], which typically first occurred around the time of cessation of breathing and before cardiac arrest, but was only performed in <50% of pigs (40 ms^−1^ = 6/12 pigs; 60 ms^−1^ = 9/24 pigs; 80 ms^−1^ = 5/12 pigs; and 100 ms^−1^ = 4/12 pigs). Due to the low occurrences, modeling for individual leg movement frequencies (e.g., left/right leg flick) was not performed. When all leg movements (flick, twitch, contract, and paddle) were combined, the faster cycle rates were shown to result in higher frequencies [*X*^2^_(3, 13)_ = 15.95, *p* = 0.0011] compared to the slower rates, with the highest number observed in the 80 ms^−1^ rate. Pairwise comparisons only identified a difference between the 60 ms^−1^ and 80 ms^−1^ (*t*_ratio_ = −3.96, *p* = 0.0037). Cycle length comparisons were not possible for leg movements due to the low number of animals displaying the behaviors.

Ear flicks were observed in approximately half of pigs (40 ms^−1^ = 5/12 pigs; 60 ms^−1^ = 11/24 pigs; 80 ms^−1^ = 9/12 pigs; and 100 ms^−1^ = 7/12 pigs), and first occurrences were observed early in the cycle (2–5 s), with no effect of cycle rate [*X*^2^_(3, 18)_ = 1.83, *p* = 0.6090]. The frequency of ear flicks did not change as a result of cycle rate [*X*^2^_(3, 30)_ = 4.14, *p* = 0.2471], and only 10 pigs (16.7%) were observed performing the behavior greater than 10 times, with two to three pigs per cycle rate. Female pigs (2.8 ± 1.5, 95% CI 0.9, 8.7) performed more ear flicks than male pigs (1.1 ± 0.6, 95% CI 0.4, 3.4; $X_{\left(1,30\right)}^{2}$ = 12.0, *p* = 0.0005] and heavier pigs were also shown to display a higher frequency of ear flicks compared to lighter pigs (coefficient = 0.36 ± 0.15, *p* = 0.0204). Head shaking was a rare behavior to be observed with only seven pigs in total displaying the behavior (40 ms^−1^ = 2/12 pigs; 60 ms^−1^ = 2/24 pigs; 80 ms^−1^ = 3/12 pigs; and 100 ms^−1^ = 0/12 pigs), and therefore preventing modeling for latencies and counts. For pigs that did perform head shaking, the maximum frequency observed was 5 (range 1–5) and latencies ranged between 2 and 95 s. Nasal discharge was seen in 21 pigs across all cycle rates and was either bloody (80 ms^−1^ = 2/12 pigs; 100 ms^−1^ = 1/12 pigs), bubbling {40 ms^−1^ = 1/12 pigs; 60 ms^−1^ = 5/24 pigs [only 1 pig in short cycle (480 s)]; 80 ms^−1^ = 3/12 pigs; and 100 ms^−1^ = 5/12 pigs}, or clear fluid [40 ms^−1^ = 4/12 pigs; 60 ms^−1^ = 6/24 pigs (2 pigs in short cycle (480 s)]; 80 ms^−1^ = 4/12 pigs; and 100 ms^−1^ = 7/12 pigs). There was no effect of cycle rate on the latency to any nasal discharge which varied widely within cycle rates [*X*^2^_(3, 21)_ = 2.33, *p* = 0.5064], however, in general, occurred later on in the cycle and following cardiac arrest. Bubbling was observed to have the highest incidences within a single pig, compared to other nasal discharge classifications (range: 1–30 occurrences from a single pig). There was no effect of cycle rate on combined counts of nasal discharge [*X*^2^_(3, 30)_ = 4.83, *p* = 0.1848]. Cycle length comparisons were not possible for ear flicks, head shaking, or nasal discharge due to the low number of animals displaying the behaviors.

Visible abdominal swelling was apparent in all pigs, with its occurrence starting before cardiac arrest in 53% of pigs (40 ms^−1^ = 6/12 pigs; 60 ms^−1^ = 12/24 pigs; 80 ms^−1^ = 8/12 pigs; and 100 ms^−1^ = 6/12 pigs). Cycle rate had no impact on the latency to visible abdominal swelling [*X*^2^_(3, 30)_ = 0.18, *p* = 0.9807] nor did cycle length (within 60 ms^−1^ rate only: 480 s or 720 s) [*X*^2^_(1, 12)_ = 1.94, *p* = 0.1641]. Swelling initially gradually increased for a period of ~30 s and then stabilized, and mild swelling was maintained until the cycles ended and recompression was initiated.

## Discussion

The primary aim of the present study was to determine, from first principles, the effectiveness of hypobaric hypoxia *via* gradual decompression to irreversibly stun pigs for application to commercial slaughter, while protecting welfare through the application of general anesthesia. We demonstrate that decompression provides an effective non-recovery process resulting in the death of 100% of weaner-grower pigs within proposed time restraints (25). Our work confirms the potential of decompression for stunning purposes and justifies subsequent study in conscious pigs. The results presented here should be considered in conjunction with the pathological assessments presented as a companion paper (41).

Our multi-disciplinary approach employing a range of physiological techniques and reflexive behavioral data revealed predictable and cumulative effects indicative of hypobaric hypoxia across a range of measures. These included physiological reductions in HR, BIS, and SpO_2_ as the cycle progressed, along with behavioral indicators such as the onset of abnormal breathing, cessation of breathing, and whole-body movements. As expected, we found overarching effects of cycle rate on several physiological and behavioral markers, with faster decompression rates eliciting shorter latencies to produce a non-recovery state. Cardiac arrest generally preceded respiratory arrest (67.2% of pigs) occurring approximately 64.3 s before the observed cessation of rhythmic breathing, and BIS values covaried with time to cardiac arrest. As expected, faster decompression rates caused earlier cardiac arrest and cessation of normal breathing. Cycle length had limited effects on all parameters of interest, indicating that we may safely shorten the hold phase of the cycle in future work. Furthermore, key parameters indicating a non-recovery state (latencies to cardiac arrest and cessation of breathing) did not appear to be directly related to thresholds in chamber pressure and the associated atmospheric oxygen equivalent (%) - all occurring below ~45 kPa, with faster decompression rates resulting in latencies near ~25 kPa chamber pressure.

The decompression rates reported in this study represent the average decompression rate during phase 1 of the decompression cycle, but it is important to note that the decompression profiles are not linear during this phase, and therefore, pigs are exposed to higher rates of decompression than the reported average targets. The LAPS^®^ system (developed by TechnoCatch LLC, USA) used in this study calculates the average decompression rate during set intervals relating to pressure thresholds, generating stepwise programming of the decompression curve (27). The fastest rates experienced occur at the very start of the cycle (progression from the start of the cycle (~101 kPa) to the first threshold of ~85 kPa). In the fastest decompression rate evaluated (100 ms^−1^), the highest absolute rates were observed, for example, ~151 ms^−1^, while for the slowest rate (40 ms^−1^) in the same step, the rate was near 70 ms^−1^. As such, animal-related outcomes may be related to the average target decompression rate plus a range of rates at each interval step which generates the shape of the curve. A recent study investigating the potential of hypobaric hypoxia *via* gradual decompression as an alternative to CO_2_ killing of rodents in laboratory demonstrated behavioral and pathological outcomes based on both the decompression rate and the shape of the decompression curve (57). The target average rates investigated here, as well as the regulated commercial broiler LAPS profile (32), are within pressure change ranges (e.g., kPas^−1^) applied to humans as part of altitude flight training, and result in a low prevalence (~9%) of self-reported concerns (e.g., aerotitis media, hyperventilation, decompression sickness, etc.) (58). However, not all of these reports may be relevant to decompression profiles employed for animal stunning due to their shorter time span (59), although self-reported pain in humans reduce with slower rates of ascent in hypobaric training (60). We found only marginal decreases in the latency of key indicators of death (cardiac arrest and cessation of breathing), with faster rates, so there is little to be gained in terms of throughput while increasing the potential for pain-related concerns [e.g., aerotitis media which has been reported in both humans (58) and mice (based on pathology) (57)]. Such pathological concerns were not observed in poultry at the average decompression rate of 127 ms^−1^ (30), however, highlighting the need for caution when extrapolating between birds and mammals. The minimal differences in indicators of death also support the exclusion of the slowest rate for future study in conscious pigs, as the prolonged onset to the cessation of breathing resulted in longer durations of both tachypnea and abnormal breathing, which in a conscious animal could potentially expose them to longer periods of respiratory distress (9, 34).

When considering the potential effects of gradual decompression for slaughter purposes, time to result in a non-recovery state is not the only important factor; carcass quality must also be minimally affected by any stunning method for it to be commercially viable. Therefore, pathological assessment (41) and the number of whole-body movements (possibly reflecting convulsive activity) are important. We observed greater repeated and prolonged whole-body movements in more pigs undergoing the slowest rate (40 ms^−1^) compared to all other rates, with the lowest number in the intermediate 80 ms^−1^ rate, again suggesting that it should not be pursued. The observed abdominal swelling is a potential concern. The latencies for swelling onset occurred approximately between 110 and 320 s post cardiac arrest, suggesting that the risk to animal welfare is minimal as the pigs had been in a sustained non-recovery state for >2 min by this time. Its impacts may be more relevant to pathology outcomes and potential carcass and meat quality effects, as the mild swelling was maintained until recompression was initiated, therefore the increased length of a cycle would result in a longer period of distention, although this did not translate to differences in pathological outcomes reported in (41).

General anesthesia was used as an essential step to protect pig welfare, however, in addition to making animal welfare assessment impossible, this introduced several potential limitations. Most notably, the latencies associated with the cessation of physiological markers indicative of death are confounded by anesthesia (which reduces HR, BIS, etc.). Although pigs under general anesthesia are normally maintained on supplementary oxygen, this would have been counter-productive to the evaluation of hypobaric hypoxia as a potential stun-to-kill method. Pigs, therefore, entered the decompression chamber in a slightly hypoxic state (as reflected by reduced baseline SpO_2_ levels), which may have important effects on the time to succumb to hypoxia and death. Hypoxic and anesthetized pigs likely succumb to hypobaric hypoxia more quickly than conscious pigs, and therefore, the timings for terminal events reported here are likely to be underestimated. Further, although a standardized anesthesia protocol was used, there was inevitably slight variation in the depth of anesthesia between individuals, as reflected by variation in BIS values (though all pigs were unresponsive on loading into the chamber and top-up anesthesia protocols were in place if required). This could account for increased inter-individual variability across our measures, which would be more limited in conscious animals. Additionally, the pigs were held in slings, and although they were breathing spontaneously, the weight of the body in the slings may have affected respiratory movements and tidal volume, again potentially influencing the susceptibility of anesthetized pigs to hypobaric hypoxia. Although the anesthesia monitors used to collect and display physiological data were essential for real-time monitoring during the experiments, they are designed to work within certain “normal” physiological ranges and environmental ranges (e.g., temperature, RH, and atmospheric pressure) [DatexOhmeda (GE) S/5 Compact Anesthesia Monitor, US; BIS™ Complete 2-Channel Monitor (Medtronic, USA)], so their output became less reliable and accurate as the pigs reached extreme values and died, as well as operating under substantial changes in chamber pressure. This led us to discount some of the monitor data for further analysis, based on unreliability. As a result, although our findings provided an essential initial step, cautious extrapolation to conscious pigs of the rates explored here on time of hypoxia and death is essential.

The physiological responses seen were generally as expected in response to hypobaric hypoxia. The heart rate increases in the early part of the decompression observed at 40 ms^−1^ were not seen in other treatments. During hypobaric hypoxia in humans, the body compensates with increased depth of respiration, increased rate of respiration, and increased heart rate in an attempt to maintain oxygen delivery to the tissues. The slower decompression rate of 40 ms^−1^ likely caused mild hypoxia initially, providing an opportunity for this compensation to happen which was not possible with faster rates. In poultry exposed to LAPS, no tachycardia is seen (26), so heart rate responses appear to vary with species and rate of decompression. In conscious animals, heart rate changes will be confounded with activity and fear responses, so these data do not allow us to predict heart rate responses in conscious animals, especially in the early part of LAPS. Increases in BIS were also seen in the early part of the decompression cycles, which may reflect some sensory stimulation (albeit in an unconscious animal) or pressure reversal effects on anesthetic depth (though these are generally attributed to hyperbaria) (61).

The inclusion of both sexes was considered crucial for this proof of principle step, and therefore, we investigated the potential of hypobaric hypoxia *via* gradual decompression on both male and female pigs. Effects of sex were noted in several physiological and behavioral measures; however, these are difficult to explain and comparisons based on sex (a secondary factor of interest) were limited in *a priori* power analysis due to the absence of relevant available data, therefore the N for sex comparisons (*n* = 6) may limit interpretation. Male pigs were found to have lower heart rates compared to females during hypobaric hypoxia, higher heart rate variability, delayed latencies to cardiac arrest and were observed to stop breathing prior to females. However, the responses of the sexes within the decompression rate and throughout the process all showed a similar pattern. Additionally, weight was not found to have an effect on statistical modeling, and therefore, weight differences between the sexes can be ruled out. Another explanation may be to apply an ethological theory, whereby male pigs in a sexually dimorphic species can be more vulnerable to morbidity and mortality [as observed in domestic pigs (62)] due to their higher energetic demands and evidence of higher oxidative stress, which in turn can impact cardiovascular health, as well as other pathologies (63, 64). In this study, however, we can only speculate, and it is important to consider that our results may not represent genuine sex differences.

## Conclusion

This study represents the first detailed exploration of the effects of hypobaric hypoxia on pigs and demonstrates the capacity of gradual decompression to reliably elicit non-recovery states within proposed commercial time constraints. Although pigs were maintained under general anesthesia, and their behavior was necessarily limited, useful information was gleaned and with concurrent physiological responses, provides a basis on which to proceed with work on conscious pigs. While the current data do not provide information with regard to the welfare consequences of hypobaric hypoxia, along with the pathological findings (41), they define the rate range of interest for decompression in further work. The acceptability of a stunning method depends on various factors including animal welfare, meat quality, cost, and consumer acceptance; all must be satisfactorily met if a new approach is to be widely adopted. Given the concerns around current stunning approaches for pigs, welfare outcomes must be particularly scrutinized, and the next phase of this work should focus on evaluating the responses of conscious pigs to intermediate decompression rate parameters and in direct comparison to a controlled atmosphere stunning with carbon dioxide.

## Data availability statement

The raw data supporting the conclusions of this article will be made available by the authors, without undue reservation.

## Ethics statement

The animal study was reviewed and approved by University of Edinburgh and SRUC Animal Welfare and Ethical Review Bodies (AWERBs, study approval refs: L325 and ED AE14-2018).

## Author contributions

JM, EB, and DM contributed to the conception, funding acquisition, and experimental design of the study. JM, MF, and EB all contributed to various levels of data collection, experimental planning, and practical work. RC, RG, and SG provided veterinary care, anesthesia induction, and maintenance for the pigs. JM and JC conducted all data processing and analysis. JM, JC, EB, and DM jointly wrote the first draft of the manuscript. All authors contributed to the manuscript revision and final editing.

## Funding

This research was jointly funded by the Department for Environment, Food and Rural Affairs (DEFRA), UK Government (Grant reference MH0154), and the Humane Slaughter Association (HSA), UK. The Roslin Institute is funded by a BBSRC Institute Strategic Program Grant BB/P013759/1. SRUC receives funding from the Scottish Government's Rural and Environment Science and Analytical Services Division (RESAS). For open access, the author has applied a Creative Commons Attribution (CC BY) license to any Author Accepted Manuscript version arising from this submission.

## Conflict of interest

The authors declare that the research was conducted in the absence of any commercial or financial relationships that could be construed as a potential conflict of interest.

## Publisher's note

All claims expressed in this article are solely those of the authors and do not necessarily represent those of their affiliated organizations, or those of the publisher, the editors and the reviewers. Any product that may be evaluated in this article, or claim that may be made by its manufacturer, is not guaranteed or endorsed by the publisher.
