# Supplementary material for: Characterizing candidate decompression rates for hypobaric hypoxic stunning of pigs. Part 1: Reflexive behavior and physiological responses
Source: Front Vet Sci. 2022 Nov 29;9:1027878. doi: 10.3389/fvets.2022.1027878 (PMC9745335; doi:10.3389/fvets.2022.1027878)
Supplement: Supplementary file 1 [file Data_Sheet_1.pdf]

Supplementary Material

1 Supplementary Figures and Tables

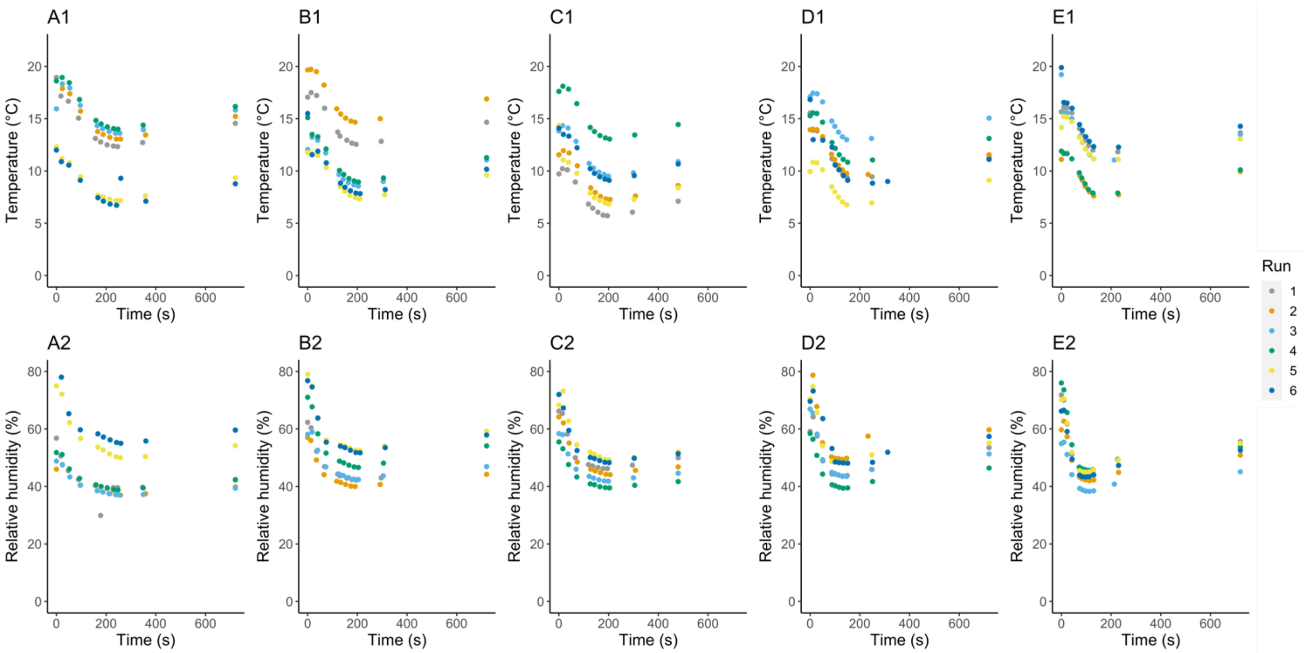

Figure S1. Temperature (°C) and relative humidity (%) recorded at target pressure (kPa) set points (101.3kPa, 84.4kPa, 67.5kPa, 50.5kPa, 33.6kPa, 30.2 kPa, 26.8kPa, 24.3kPa, 23.4kPa, 21.7kPa and 20.9kPa) relative to cycle time (s) of each of the six cycle runs undertaken for the five decompression treatment profiles: A1 & A2) 40ms^{-1}, B1 & B2) 60ms^{-1}, C1 & C2) 60ms^{-1} (short), D1 & D2) 80ms^{-1} and E1 & E2) 100ms^{-1}.

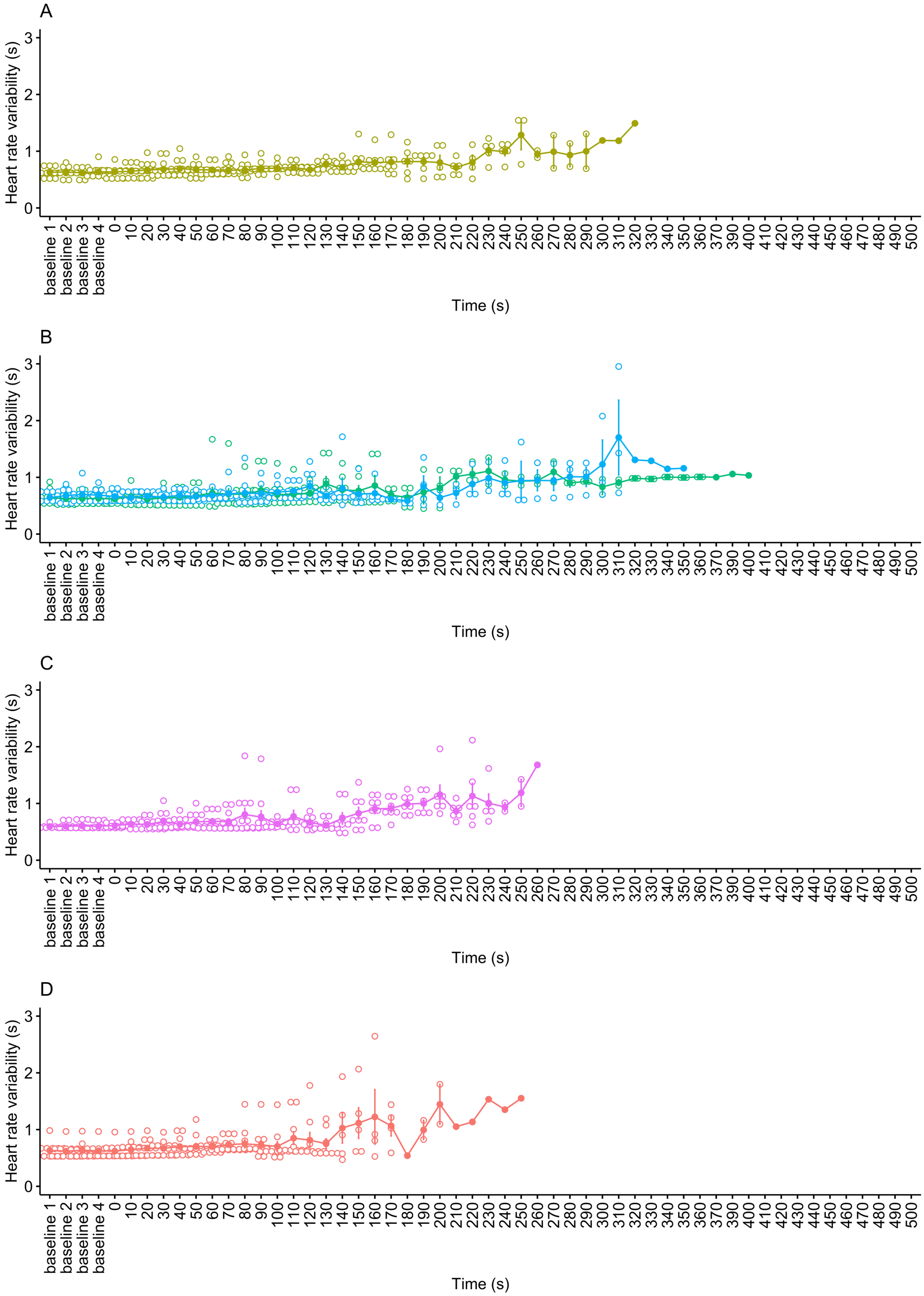

Figure S2. Mean ±SE heart rate variability (i.e., the length of time between heart beats (QRS complexes) in seconds (s)) over time for each decompression treatment rate: A) 40ms^{-1} (yellow); B) 60ms^{-1} long/720s cycle (green) and 60ms^{-1} short/480s cycle (blue); C) 80ms^{-1} (pink) and D) 100ms^{-1}

(red). Mean is represented by solid dot with error bars for SE, with hollow dots representing individual raw data points. The x axis has been limited to the baseline period and 500s into the decompression cycles only. Individual HRV data was omitted post cardiac arrest, defined by permanent loss of mechanical cardiac activity, represented by the time at which the pulse volume contour on the pulse plethysmograph became permanently imperceptible.
